# Transcriptomic signaling pathways involved in a naturalistic model of inflammation-related depression and its remission

**DOI:** 10.1038/s41398-021-01323-9

**Published:** 2021-04-06

**Authors:** Marie-Pierre Moisan, Aline Foury, Sandra Dexpert, Steve W. Cole, Cédric Beau, Damien Forestier, Patrick Ledaguenel, Eric Magne, Lucile Capuron

**Affiliations:** 1 Univ. Bordeaux, INRAE, Bordeaux INP, NutriNeuro, UMR 1286, Bordeaux, France; 2 Division of Hematology-Oncology, Department of Psychiatry & Biobehavioral Sciences and Department of Medicine, UCLA School of Medicine, Los Angeles, CA USA; 3 Service de Chirurgie Digestive et Pariétale, Clinique Tivoli, Bordeaux, and Clinique Jean Villar, Bruges, France

**Keywords:** Predictive markers, Psychology

## Abstract

This study aimed at identifying molecular biomarkers of inflammation-related depression in order to improve diagnosis and treatment. For this, we performed whole-genome expression profiling from peripheral blood in a naturalistic model of inflammation-associated major depressive disorder (MDD) represented by comorbid depression in obese patients. We took advantage of the marked reduction of depressive symptoms and inflammation following bariatric surgery to test the robustness of the identified biomarkers. Depression was assessed during a clinical interview using Mini-International Neuropsychiatric Interview and the 10-item, clinician-administered, Montgomery–Asberg Depression Rating Scale. From a cohort of 100 massively obese patients, we selected 33 of them for transcriptomic analysis. Twenty-four of them were again analyzed 4–12 months after bariatric surgery. We conducted differential gene expression analyses before and after surgery in unmedicated MDD and non-depressed obese subjects. We found that TP53 (Tumor Protein 53), GR (Glucocorticoid Receptor), and NFκB (Nuclear Factor kappa B) pathways were the most discriminating pathways associated with inflammation-related MDD. These signaling pathways were processed in composite *z*-scores of gene expression that were used as biomarkers in regression analyses. Results showed that these transcriptomic biomarkers highly predicted depressive symptom intensity at baseline and their remission after bariatric surgery. While inflammation was present in all patients, GR signaling over-activation was found only in depressed ones where it may further increase inflammatory and apoptosis pathways. In conclusion, using an original model of inflammation-related depression and its remission without antidepressants, we provide molecular predictors of inflammation-related MDD and new insights in the molecular pathways involved.

## Introduction

Over the past two decades, compelling evidence has emerged to highlight the role of inflammation in the onset and perpetuation of major depressive disorders (MDD). Epidemiological and clinical studies have repeatedly shown the high prevalence of depressive symptoms in chronic inflammatory disorders. Furthermore, chronic treatment with the pro-inflammatory cytokine, interferon-alpha, in medically ill patients was found to be responsible for the development of major depression in over 30% of patients^1,2^. Consistent with these data, a recent meta-analysis confirmed that MDD is associated with elevated levels of pro-inflammatory cytokines, such as interleukin (IL)-6 and tumor necrosis factor (TNF)-α^3^. Additionally, genetic studies have revealed the role of immune genetic variants in MDD^4^. Interestingly, inflammation has been recently hypothesized to also have a role in antidepressant treatment resistance, as patients presenting signs of systemic low-grade inflammation display a poor response to classical antidepressants, such as selective serotonin reuptake inhibitors^5^. Thus, a better understanding of the molecular mechanisms underlying inflammation-related depression is needed. In particular, the identification of biomarkers specific to this depression subtype is required for improving its diagnosis and treatment.

Transcriptomic analysis from peripheral blood has successfully been used to reveal biological pathways linked to MDD^6,7^. A recent study examined the implication of specific molecular pathways in inflammation-related depression by measuring self-reported depressive symptoms 2 h after endotoxin injection in healthy subjects^8^. However, hypothesis-free transcriptomic analyses have not been performed specifically in diagnosed inflammation-associated MDD patients. To address this issue, we used a naturalistic model of inflammation-related depression represented by comorbid depression in obese patients. Indeed, obesity is a chronic medical condition characterized by a low-grade inflammatory state associated with an increased prevalence of depression^9^. Remarkably, systemic inflammation, reflected by increased levels of C-reactive protein levels, predicts obesity-related depressive symptoms better than metabolic health per se^10^. Interestingly, bariatric surgery-induced weight loss was found to correlate with a reduction of both inflammation and depressive symptoms^11,12^.

In this study, we sought to identify transcriptional control pathways associated with inflammation-related depression in unmedicated obese patients. For this purpose, gene expression profiling of peripheral blood was performed and compared between depressed and non-depressed severely obese patients before and after bariatric surgery, with the hypothesis that these biological pathways would be changed after surgery-induced weight loss and subsequent decrease in inflammation. Upstream regulator analyses were employed to uncover the molecular signaling pathways that differentiate depressed from non-depressed subgroups of patients. Finally, the most discriminating signaling pathways identified were processed in composite *z*-scores of gene expression in order to be used as biomarkers predicting depressive symptoms.

## Methods

### Patients

One hundred severely or morbidly obese patients [body mass index (BMI) > 35 kg/m^2^] awaiting bariatric surgery were recruited from the services of digestive and bariatric surgery at two private clinics (Tivoli and Jean Villar clinics, Bordeaux, France). Patients were scheduled to receive either a sleeve gastrectomy or a gastric bypass. Patients were chosen on the basis of their levels of depressive symptoms in order to cover a range from non-depressed to clinically depressed patients. Seventy-three percent of patients (*n* = 24) were followed up at 4–12 months after surgery (mean time at follow-up 6.5 months). The variability in the follow-up interval times is due to the patient not coming at the first appointments. Exclusion criteria were: age >65 years old; acute or chronic inflammatory conditions (other than obesity); current treatment with antidepressants or any other psychotropic drug; current diagnosis of psychiatric disease (except for major depression); and/or severe medical illness including neurological disorders and cancer. Patients using anti-inflammatory medications (checked at recruitment and at medical consultation) were excluded. The study was approved by the local Committee for the Protection of Persons (Bordeaux, France). All patients provided written informed consent after reading a complete description of the study.

### Clinical evaluation

DSM criteria for current major depression were determined using the Mini-International Neuropsychiatric Interview (MINI) administered during a semi-structured interview by trained raters at baseline and after bariatric surgery^13^. Concomitantly, the intensity of depressive symptoms was assessed using the 10-item, clinician-administered, Montgomery–Asberg Depression Rating Scale (MADRS)^14^.

### Genome-wide transcriptional profiling

Blood samples were collected twice, before (*n* = 33) and after surgery (*n* = 24) in study participants. Five ml of venous blood was collected per patient on vacuum tubes (PAXgene Blood RNA system; PreAnalytiX GmbH, Hombrechtikon, Switzerland). The samples were maintained at room temperature for >2 h as required for stabilization of RNA and then kept at −20 °C until RNA extraction. Total RNA was extracted using the PAXgene Blood RNA Kit (Qiagen, Courtaboeuf, France) according to the manufacturer’s protocol. The quality of the total RNA was assessed using RNA Nano chips on a Bioanalyser 2100 (Agilent, Boeblingen, Germany). All samples had an RNA Integrity Number (RIN) score >8.0. RNA concentrations were measured with a Nanodrop spectrophotometer.

Gene expression profiles of the 57 samples (33 before and 24 after surgery) were analyzed in a single batch at the GeT‐TRiX facility (GenoToul, Génopole Toulouse, France) using Agilent SurePrint G3 Human GE V2 8x60K (Design 039494) microarrays following the manufacturer’s instructions. Microarray data and experimental details are available in NCBI’s Gene Expression Omnibus^15^ and are accessible through GEO Series accession number GSE99725.

### sPLS regression

Sparse Partial Least Square (sPLS) regression^16^ using the R package, mixOmics (http://www.mixOmics.org) was performed to select the expressed genes that correlated the best to MDD diagnosis or MADRS items. From the 2000 probes found highly correlated with a correlation coefficient *r* > 0.5, 1474 unique genes were identified.

### Differential gene expression analyses

Raw data (median of pixel intensity) were analyzed using R (www.r-project.org, R v. 3.1.2) Bioconductor packages (www.bioconductor.org, v 3.0^17^) as described in GEO entry GSE99725. Briefly, raw data were filtered, log2 transformed, corrected for bath effects (washing and labeling serials), and normalized using the quantile method^18^. A first exploratory and statistical analysis showed a possible correlational structure among gene expression, which could negatively impact the multiple testing procedures. We applied the FAMT method^19^ to reduce the dependence structure. A model was fitted using the limma lmFit function^20^ considering “patients” as a blocking factor for the time pairs using a duplicate correlation function. A correction for multiple testing was then applied using the Benjamini–Hochberg procedure^21^ for false discovery rate (FDR). Probes with FDR ≤ 0.05 were considered differentially expressed between conditions.

### Impact of blood cell proportions in gene expression profiles

We assessed whether the observed gene expression changes in the different analyses were related to changes in cell proportions in the blood samples using the Cell-type Computational Differential Estimation CellCODE R package^22^.

### Transcript origin analysis

To identify specific cell types that contributed to the empirically observed differences in gene expression, we applied transcript origin analysis^23^ to separate lists of upregulated and downregulated genes. TOA tested for significant overrepresentation of genes preponderantly expressed by a specific subset of blood cells (i.e., monocytes, dendritic cells, CD4-positive T cells, CD8-positive T cells, B cells, or NK cells) using cell-specific reference transcriptomes derived by flow cytometric isolation^23,24^.

### Transcriptional pathways identification, comparison analysis, and composite *z*-scores analyses

The biological pathways involved in MDD were predicted from lists of differentially expressed genes using the “Upstream Regulator Analysis” from Ingenuity Pathway Analysis (IPA, Qiagen, USA).

To calculate the composite z-scores representing a given signaling pathway, we first extracted the list of target genes for a given transcription factor from ChIP-seq experiments available in ChEA 2016, an integrative database of public ChIP-seq data (available in http://amp.pharm.mssm.edu/Enrichr/). Then, from the list of target genes for a given transcription factor, we selected the ones that were expressed in our data set. For each of the patients, the expression data for each gene was first standardized by calculating a *z*-score, *z*-score = $\frac{{x - \mu }}{\sigma }$, where *x* is the gene expression data, *μ* the mean of the gene expression for the population (all patients of the study) and σ the standard deviation for the population. For each patient, the composite *z*-score for a given transcription factor was then calculated as the mean of the target genes’ *z*-scores.

### Cytokines measures in serum

High-sensitivity C-reactive protein (CRP) was measured from fasting blood samples as described in ref. ^10^ using an ELISA kit (Millipore, Billerica, MA). Similarly, high-sensitivity Interleukin-6 (IL-6) was measured from an ELISA kit (R&D Systems, Minneapolis, MN) according to manufacturer instructions.

### Statistics

Two-tailed *t*-tests, ANOVA, and Multivariate regression analyses were done with the software Statistica and the graphs designed with GraphPadPrism. A *p*-value of *p* < 0.05 was considered significant.

## Results

### Characteristics of participants

The general characteristics of the participants are described in Table 1. Since study participants were chosen at baseline to cover a range from non-depressed to clinically depressed subjects, the percentage of patients with MDD in the present study (45.45%) is higher than what is generally found in the obese population. There were no statistical differences in terms of age, gender, BMI (before or after surgery), CRP, IL-6 or interval time after surgery between non-depressed versus depressed patients (unpaired *t*-tests except Fisher’s exact test for % women). As expected, MADRS scores were significantly higher in depressed patients at baseline (*p* < 0.0001) and remained significantly higher after surgery (*p* = 0.0068) although all patients were no more depressed. The number of patients that were lost after follow-up was balanced between the 2 groups, 4 depressed and 5 non-depressed patients. These patients did not come to the follow-up medical consultation probably by lack of interest but not because of health complications. BMI was strongly reduced after surgery in the 24 patients with available data at follow-up either as a whole or within depressed or non-depressed subgroups (paired *t*-test *p* < 0.0001 for each analysis). Similarly, the percentage of patients with MDD was significantly decreased after surgery compared to baseline (from 45.45 to 0%, Fisher exact test *p* < 0.0006) as well as the MADRS scores in the depressed subgroup (paired *t*-test, *p* < 0.0002).

**Table 1:** Characteristics of study participants.

	MDD baseline *n* = 15	Non-MDD baseline *n* = 18	MDD post-surgery *n* = 11	Non-MDD post-surgery *n* = 13
Age	38.13 ± 12.39	38.83 ± 11.14		
Women, *n* (%)	13 (86.7%)	17 (94.4%)		
BMI, kg/m^2^	41.82 ± 4.28	42.24 ± 5.85	30.86 ± 4.84	31.62 ± 5.94
MADRS score	19.00 ± 3.87^a^	6.56 ± 3.59	6.36 ± 5.09^a^	2.08 ± 3.29
CRP (mg/l)	10.51 ± 7.58	7.99 ± 5.34		
Il-6 (pg/ml)	2.92 ± 2.13	3.45 ± 2.17		
Post-surgery interval	5.97 ± 2.99	6.92 ± 2.65		

All data are mean ± SD unless indicated.

*BMI* body mass index, *CRP* plasma C-reactive protein, *IL-6* plasma interleukin-6.

^a^Indicates a statistical difference between MDD and non-MDD patients by *t*-test.

### Gene expression analyses

**Fig. 1:**
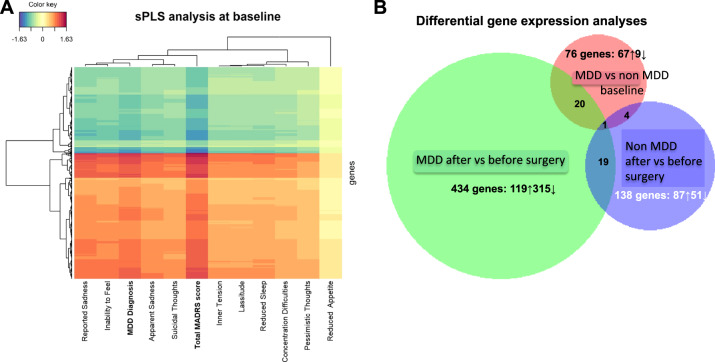
Gene expression analyses at baseline and after bariatric surgery in MDD and non-depressed patients. **A** Heatmap of the results of the sparse least square analysis (sPLS) on the 33 patients at baseline showing the strength of correlation between the 2000 gene probes the most correlated to depression (color code at the top left); each line corresponds to a gene probe and in each column a phenotypic value of depression. **B** Venn diagrams of the three differential expression analyses showing significantly differentially expressed genes.

As a first exploration of the peripheral blood gene expression data from the 33 obese patients at baseline, we performed an sPLS regression analysis with the variables related to depressive symptoms. The heatmap (Fig. 1A) illustrates the strength of correlation between the 2000 most correlated probes and the variables related to depressive symptomatology. Strong correlations with gene expression were found with total MADRS score and MDD diagnosis. Not surprisingly, the correlation was the weakest with the MADRS item measuring reduced appetite. When the selected variable was the total MADRS score, the 2000 most correlated probes corresponded to 1474 unique genes, 428 downregulated and 1046 upregulated. When this gene data set was submitted to IPA, the top canonical pathway identified was the Glucocorticoid Receptor (GR) signaling pathway (*p*-value 2.13 × 10^−5^ with an overlap of 12.5%), while the top upstream regulators were cystatin D (CST5), tumor protein 53 (TP53) and hepatocyte nuclear factor4 (HNF4A) (*p* = 1.09 × 10^−8^, *p* = 9.8 × 10^−8^ and *p* = 6.10 × 10^−7^, respectively). The IPA analysis could not predict whether these transcription factors were activated or inhibited in patients.

Then, we performed 4 differential gene expression analyses where we compared the gene expression of MDD to non-depressed patients at baseline, MDD patients after versus before bariatric surgery, non-depressed patients after versus before surgery, and lastly, MDD versus non-depressed (as they were at baseline) after surgery.

At a false discovery rate of 5%, the three first analyses provided a number of differentially expressed genes (DEG) as illustrated in Fig. 1B, with a Venn diagram proportional to the number of DEG. For the later analysis (MDD versus non-MDD after surgery), no significant DEGs were detected as expected since patients were no longer depressed in either group. We found no significant changes in immune cell subtypes’ proportion in the different analyses with significant DEG using CellCODE (Supplementary Table 1). For the MDD versus non-MDD comparison at baseline, the transcript origin analysis revealed that upregulated transcripts derived preferentially from NK cells, CD4^+^ T cells, and CD8^+^ T cells. For the MDD after versus before surgery comparison, the upregulated transcript derived from monocytes and the downregulated genes from monocytes, dendritic cells, and NK cells. For the non-MDD after versus before surgery comparison, the upregulated transcripts originated preferentially from CD4^+^ T cells and CD8^+^ T cells and the downregulated genes from NK cells. These results are presented in Supplementary Fig. 1. The list of DEG for each analysis, and the corresponding fold change, are presented in Supplementary Table 2.

### Upstream regulator analyses

**Fig. 2:**
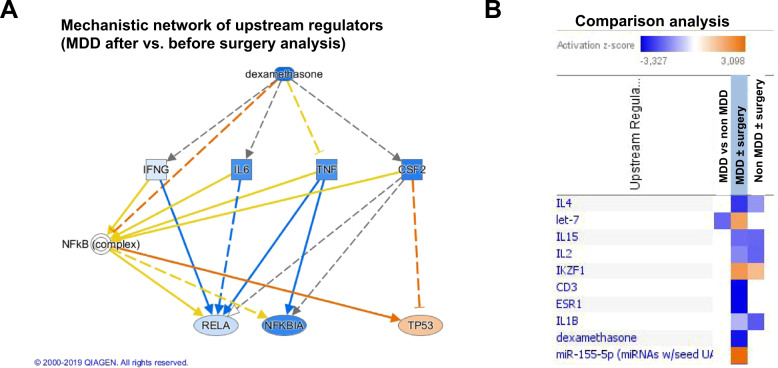
Upstream regulator analyses using Ingenuity Pathway Analysis. **A** Mechanistic network connecting upstream regulators found in the differential expression analysis between MDD patients after versus before surgery. **B** Comparison analysis of upstream regulators in the three differential expression analyses. Upstream regulators are sorted by activation z-score with color code at the top of the figure.

To identify the transcriptional pathways associated with MDD in these patients, we perform upstream regulator analyses from each list of DEG using IPA. The lists of upstream regulators for each analysis are provided in Supplementary Table 3. At a cutoff *p*-value of overlap *p* < 0.001, only one upstream regulator, TP53, was significant for the MDD versus non-MDD at baseline analysis, with 17 downstream target genes. For the MDD after versus before surgery analysis, 67 upstream regulators were detected, the three most significant were, respectively, dehydrotestosterone (*p* = 4.25 × 10^−7^, an androgen receptor agonist), TP53 (*p* = 1.6 × 10^−6^), and dexamethasone, a specific glucocorticoid receptor agonist (*p* = 2.84 × 10^−6^). TP53 and dexamethasone showed the highest number of downstream target genes with 67 and 71 target genes, respectively. Interestingly, a mechanistic network was revealed that linked dexamethasone and TP53 along with other upstream regulators of this data set (Fig. 2A). The NFκB (Nuclear Factor kappa B) complex appears as an important hub between dexamethasone and TP53 in this network. For the non-MDD after versus before surgery analysis, 7 upstream regulators were found significant but all of them with a low number of target genes, GATA1 (GATA binding protein 1) having the highest number with 9 target genes. To identify the upstream regulators that were in common or different between MDD and non-depressed patients and explaining the changes after surgery, a comparison analysis was performed (Fig. 2B). This analysis highlighted that among the 10 regulators sorted by activation *z*-score, several cytokines pathways (IL-4, IL15, IL2, IL-1β) were downregulated in both MDD and non-depressed patients after surgery. Remarkably, dexamethasone was found downregulated in the MDD after versus before surgery analysis only.

### Transcriptomic pathway biomarkers of inflammation-related depression

**Fig. 3:**
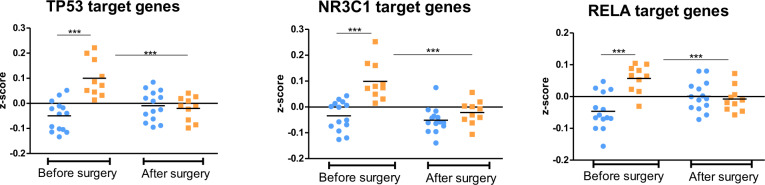
TP53, NR3C1, and RELA pathways differentiate MDD (orange symbols) from non-depressed patients (blue symbols) at baseline and are normalized after surgery. Two-way ANOVA followed by Bonferroni post hoc tests, ***p < 0.001.

The upstream regulator analyses above, in accordance with the sPLS regression, pinpointed dexamethasone/GR, NFκB, and TP53 as the most important and convergent transcriptional regulators associated with MDD. To assess whether these pathways could serve as biomarkers predicting inflammation-related depression in our sample, we calculated for each of these regulators a composite *z*-score of gene expression using all their target genes, identified from ChIP-seq experiments and expressed on the microarrays (see “Methods” section for details). The lists of target genes representing NR3C1 (for dexamethasone/GR), RELA (for NFκB), and TP53 pathways and used to calculate the composite *z*-scores are given in Supplementary Table 4. Results from a two-way ANOVA indicated that MDD patients could be differentiated from non-depressed patients at baseline by the composite *z*-scores of TP53, NR3C1, and RELA, which were normalized after surgery when patients were no longer depressed (Fig. 3) (for each graph two-way ANOVA interaction (MDD x Surgery) *p* < 0.01, Bonferroni post hoc tests: MDD versus non-depressed patients before surgery *p* < 0.001, MDD versus non-depressed patients after surgery *p* = ns, MDD patients before surgery versus after surgery *p* < 0.001).

**Fig. 4:**
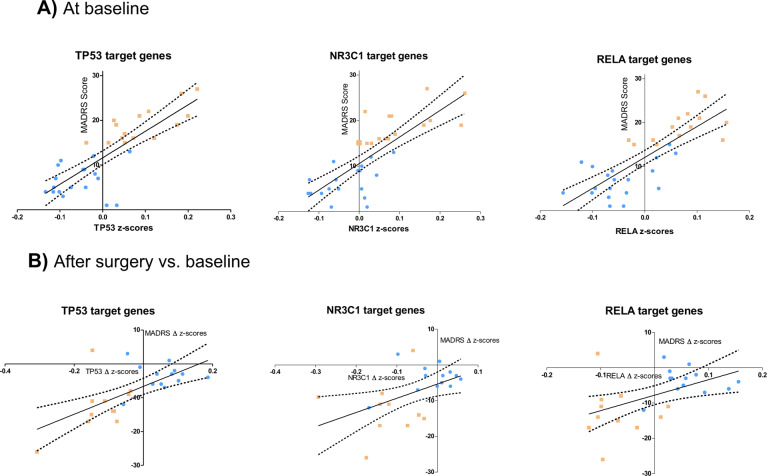
TP53, NR3C1, and RELA pathways predict the intensity of inflammation-related depressive symptoms at baseline (**A**) and their decrease after bariatric surgery (**B**). Multivariate linear regression analyses. MDD patients (orange symbols), non-MDD patients (blue symbols).

Furthermore, we tested by linear regression analysis whether these composite *z*-scores could predict the intensity of depressive symptoms at baseline using total MADRS scores (Fig. 4A). Indeed, highly significant linear relationships were detected for each of the three pathways. TP53 showed the strongest relationship (*β* = 0.80, *F*_(1,31)_ = 56.8, *p* < 10^−7^), followed by NR3C1 (*β* = 0.79, *F*_(1,31)_ = 50.3, *p* < 10^−7^) and RELA (*β* = 0.78.5, *F*_(1,31)_ = 49.8, *p* < 10^−7^). These associations remained significant after adjusting for gender, age, and BMI (Supplementary Table 5). Using the same composite *z*-scores in regression analyses with BMI, we found no relationship (Supplementary Fig. 2) suggesting the specificity of the transcriptomic biomarkers for depressive symptoms. Since depressive symptoms were considerably reduced in most obese patients after surgery, we hypothesized that the difference in composite *z*-scores before and after surgery should predict the decrease in MADRS scores from baseline to post-surgery. Consistent with this hypothesis, multivariate linear regression analyses showed a significant linear relationship for the three transcription factor composite *z*-scores (*β* = 0.65, *F*_(1,22)_ = 16.4, *p* < 0.001 for TP53, *β* = 0.47, *F*_(1,22)_ = 6.34, *p* < 0.02 for NR3C1 and *β* = 0.49, *F*_(1,22)_ = 7.19, *p* < 0.015 for RELA) (Fig. 4B).

## Discussion

Low-grade-inflammation in obesity originates primarily from the adipose tissue, in which immune cells accumulate and secrete inflammatory factors^9,25^. The resulting systemic inflammation is thought to contribute to increased neuroinflammatory processes that subsequently drive behavioral alterations, including depressive symptoms^11^. In the present study, we used this model of inflammation-related depression to reveal the transcriptomic pathways involved, taking advantage of the marked reduction of depressive symptoms and inflammation following bariatric surgery. Our transcriptomic and bioinformatic analyses identify TP53, NR3C1, and NFκB as the most important upstream regulators predicting depression in obese patients. Indeed, NR3C1/Glucocorticoid Receptor and TP53 signaling pathways were revealed as linked to depressive symptoms by an sPLS analysis using the 33 patients at baseline, and the involvement of these two upregulators were confirmed in the differential gene expression analysis comparing post-surgery versus baseline in MDD patients. Regarding NFΚB, it was first identified as an important intermediary in a mechanistic network including NR3C1 and TP53.

TP53 is known as a tumor suppressor, pro-apoptotic protein, which levels are altered in response to cellular stress. Increased apoptotic stress was reported previously in animal models of stress-induced depression and in MDD patients^26,27^. For example, a transcriptomic analysis of postmortem prefrontal cortex tissues from patients with a history of MDD revealed altered expression of apoptosis factors together with increased cytokines expression^28^. The importance of the TP53 pathway itself was described in an animal model of trauma by transcriptional analysis from blood, amygdala, and hippocampus^29^, and in a genetic study where DNA polymorphisms within the TP53 gene were found associated with MDD in a Slovak population^30^. Interestingly, TP53 was also shown to be increased in the adipose tissue of obese mice and responsible for cytokine production and senescence-like changes thereby contributing to insulin resistance^31^. Our finding that the TP53 pathway is involved in inflammation-related depression in obese individuals is consistent with these data. It is tempting to postulate that TP53 induces the secretion of pro-inflammatory factors in adipose tissue, which in turn triggers neuroinflammation and subsequent behavioral alterations.

Since MDD patients were not different from non-depressed patients in terms of BMI, it is very likely that other factors besides adiposity explain increased TP53 and NFκB signaling in MDD patients. Our analysis put forward NR3C1/GR as one possible factor since it came out in both sPLS and differential gene expression analyses as an important upstream regulator associated with MDD. Furthermore, in the comparison analysis, dexamethasone appeared as a significant upstream regulator in obese MDD patients but not in obese non-depressed patients. Evidence from the literature strongly suggests a role for GR in the development of MDD and its related neurobiological disturbances. Indeed, MDD is often preceded by a history of stress and chronically elevated glucocorticoid levels, which contribute to reduced hippocampal volume and impaired hippocampal neurogenesis commonly found in MDD^32^. Recently, GR signaling was found as the most significant pathway associated with trauma-related individual differences in a preclinical model as revealed by gene expression analysis. It was also the most significant transcription factor convergent across blood and brain tissues. Furthermore, a translational study discovered that GR sensitivity had a crucial role in antidepressant treatment response in MDD patients and in a mouse model of depression^33^. As for inflammation-related depression, a recent study examined candidate transcriptomic pathways contributing to depressed mood in healthy subjects treated acutely with endotoxin. NFκB and CREB (cAMP Responsive Element Binding Protein) signaling were found increased in subjects who developed depression but GR signaling was decreased^8^. This study confirmed earlier reports showing for example that activation of inflammatory processes in MDD is associated with a reduced GRα/β expression ratio in monocytes^34,35^ or that dexamethasone-stimulated gene expression in peripheral blood is lower in depressed patients than in healthy subjects^36^. Recently, decreased GR mRNA together with increased FKBP5 mRNA levels were found in drug-free depressed and treatment-resistant patients compared to healthy controls or antidepressant responsive patients, suggesting GR resistance^37^. Although these data may seem contradictory to ours for GR, it could be explained by the anti and pro-inflammatory actions of glucocorticoids, which depend on the timing of exposure^38^. Indeed, exposure to glucocorticoids prior to an immune challenge primes the neuroinflammatory responses in rodents^39^ as well as in human hippocampal progenitor cells^40^. Along this line, we can hypothesize that obese patients with depressive symptoms may have been exposed to more stress and elevated glucocorticoid levels before developing obesity, which would result in increased inflammation during fat accumulation. In support of this hypothesis, we showed in a previous study that patients who developed MDD during chronic interferon-α treatment displayed, as soon as the first administration of the cytokine (at the time where they were not depressed yet), an exaggerated ACTH and cortisol responses, suggestive of a sensitized stress response system^41^.

Interaction between GR and TP53 has been reported in neuronal cell cultures where dexamethasone through GR enhances TP53 activity by increasing its nuclear translocation and transcriptional activity^42^. Induction of TP53 by dexamethasone was also found in cultured tenocytes and in tendon biopsies from patients treated with dexamethasone^43^ supporting the role of GR in TP53 induction in vivo.

Besides these three main pathways, we found that several cytokines pathways were also reduced after surgery in both MDD and non-MDD patients (e.g., IL-4, IL-6). Of note, the TNF pathway was found significantly reduced in MDD patients after surgery, which is interesting in view of the efficiency of the anti-TNF agent infliximab to reduce depressive symptoms in antidepressant treatment-resistant patients^44^. HNF4A was also an interesting candidate from this study, being associated with glucose and lipid metabolism as well as with inflammation-related depression and antidepressant treatment^44^. Although the HNF4A pathway was detected in the sPLS analysis, we found no change in this pathway from the differentially expressed gene analyses may be due to lack of statistical power.

A number of studies have utilized gene expression profiles to investigate the determinants of MDD. Although few associated genes are found in common across studies, all of them implicate immune pathways, such as NFκB^45–47^, IL-6^48^, IFN-α^48–50^, oxidative stress, or apoptosis^28,47,51^ signaling pathways. These convergent findings confirm that transcriptomic pathways represent stronger biomarkers than individual genes, probably due to statistical issues, effects of cellular heterogeneity and temporal dynamics, as well as multifactorial gene regulation, as discussed before for stress genomics^52^, and heterogeneity of depression types. Indeed, the present transcript origin analyses implicated lymphoid lineage cells (particularly CD4^+^ T cells, CD8^+^ T cells, and NK cells) as primary cellular mediators of differential gene expression in MDD versus non-depressed controls under baseline conditions, as well as pre- to post-surgery transcriptome changes in non-depressed individuals. In MDD patients, by contrast, pre- to post-surgery changes were dominated by transcripts typically expressed in myeloid lineage monocytes and dendritic cells (as well as NK cells). These data suggest the potential for distinct immunoregulatory dynamics in MDD (e.g., due to differential activity of the glucocorticoid signaling system as noted above).

In most studies, the transcriptomic pathways are identified from the differentially expressed genes by bioinformatic prediction based on transcription binding motifs using various softwares (e.g., DAVID^53^, TELiS^54^) or literature curation (IPA). Here, we used a new approach relying on ChIP-seq experiments. By this method, we believe that we captured a closer reflection of the transcriptional pathways involved. Indeed, the composite *z*-score of gene expression representing the pathways of TP53, GR, and RELA was highly predictive of MADRS scores in our patient sample. Furthermore, the same composite *z*-scores were also predictive of the remission of depressive symptoms after bariatric surgery, supporting the validity of these biomarkers.

The following limitations of our study must be considered. The main one resides in the low number of patients, especially after bariatric surgery. Another limitation is that the findings may be restricted to obesity-related depression and not relevant to all inflammation-related depressions. Clearly, replication of these findings in larger cohorts and in non-obese patients with inflammation-associated depression is needed. The variability in the interval times after surgery between patients is another difficulty but as these data did not differ on average between depressed and non-depressed patients, it had no direct implication on the results presented here. The mechanism of GR over-activation versus GR resistance in inflammation-related depression remains an open question that requires further investigation. Finally, we focused on the main transcriptional pathways detected in the study while many other pathways were found significant and others may have been missed by lack of statistical power.

In conclusion, this study highlights the importance of TP53, NFκB, and GR signaling pathways in a clinical model of inflammation-related MDD and their potential as predictive biomarkers to identify individuals vulnerable to this type of depression. The present data provide insights of the molecular mechanisms involved in inflammation-associated depression that can be used to improve diagnosis and treatment.

## Supplementary information

Supplemental Figures and Tables
