# Supplementary material for: Transcriptomic signaling pathways involved in a naturalistic model of inflammation-related depression and its remission
Source: Transl Psychiatry. 2021 Apr 6;11:203. doi: 10.1038/s41398-021-01323-9 (PMC8024399; doi:10.1038/s41398-021-01323-9)
Supplement: Supplementary file 1 — Supplemental Figures and Tables [file 41398_2021_1323_MOESM1_ESM.pdf]

Supplementary Figure 1:Transcript Origin Analyses

B: B cells, CD8+ T: CD8 positive T cells, CD4+ T: CD4 positive T cells, NK: Natural Killer cells, pDC: peripheral Dendritic Cells. * p<0.05; ** p<0.01

A) MDD vs. non-MDD at baseline

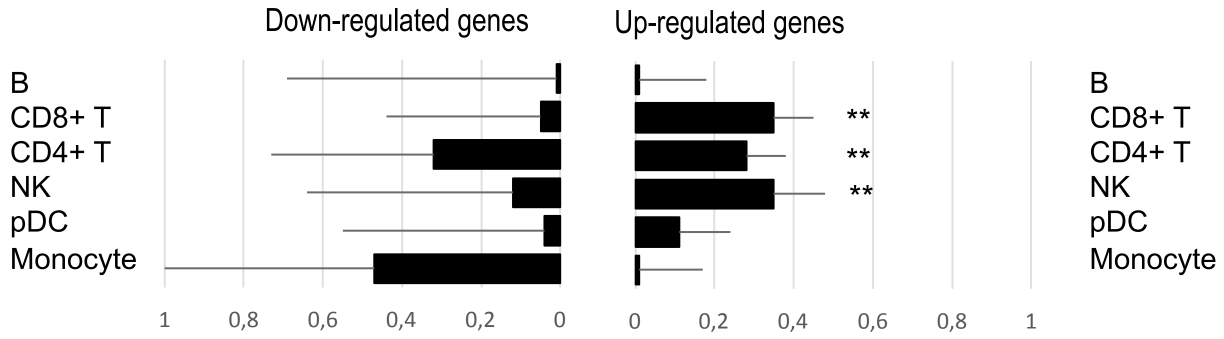

B) MDD after vs. before surgery

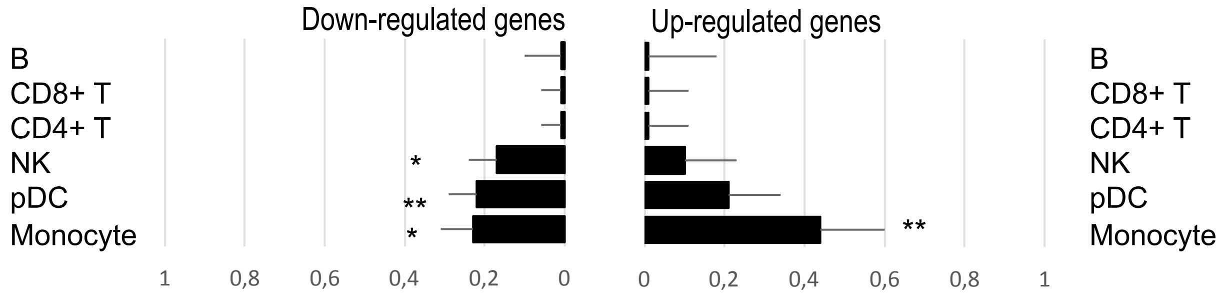

C) Non MDD after vs. before surgery

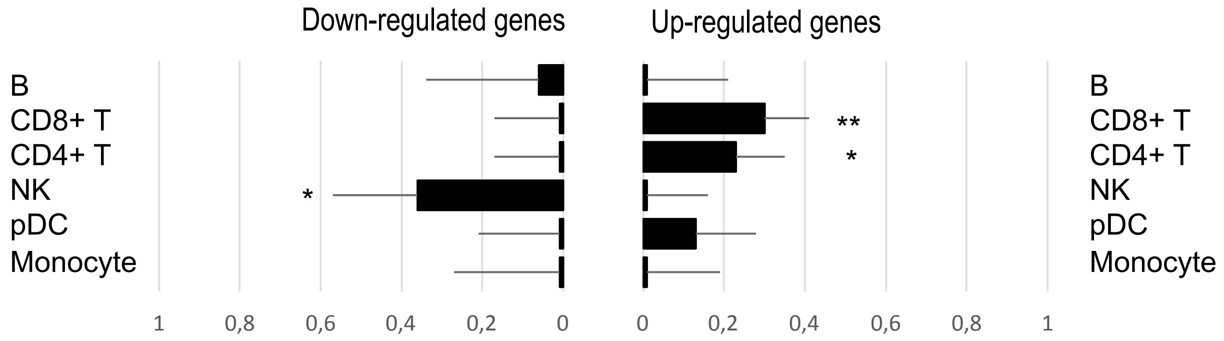

Supplementary Figure 2: TP53, RELA and NR3C1 pathways do not predict the body mass index of patients. Multivariate linear regression analyses. MDD patients (orange symbols), non MDD patients (blue symbols)

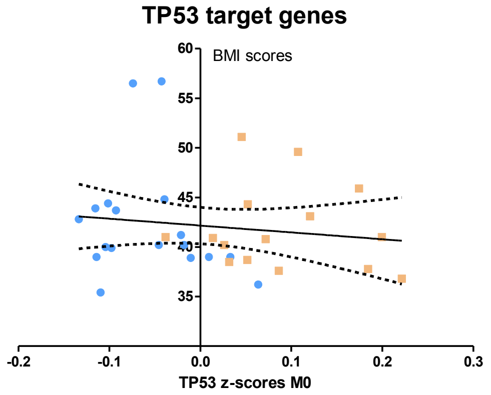

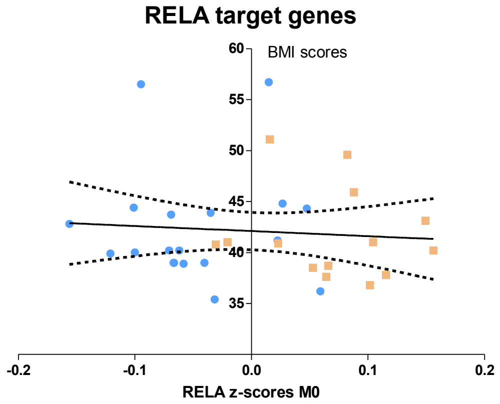

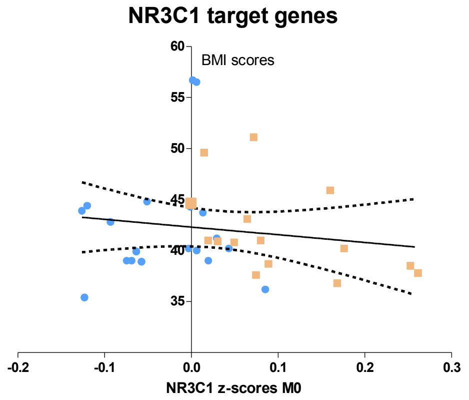

Supplementary Table 1: CellCode analysis

p value	MDD vs non MDD baseline	MDD after vs before surgery	Non MDD after vs before surgery	MDD vs non MDD after surgery
**Neutrophil**	0,09	0,2	0,79	0,34
**Tcell**	0,51	0,99	0,33	0,99
**Monocyte**	0,97	0,09	0,91	0,04
**Bcell**	0,7	0,82	0,8	0,21
**NKcell**	0,15	0,37	0,88	0,75
**Megakaryocyte**	0,9	0,84	0,78	0,61
**Erythrocyte**	0,69	0,16	0,05	0,62

Supplementary Table 2: Lists of differentially expressed genes for each analysis

MDD vs non-MDD baseline		MDD after vs. before surgery		Non-MDD after vs. before surgery	
GeneSymbol	log2FC	GeneSymbol	log2FC	GeneSymbol	log2FC
MAFF	-1,26	RASA1	-1,715	HIVEP2	-1,611
STAMBP	-1,06	BCL2	-1,593	BBS7	-1,301
CASS4	-0,92	MAP4	-1,578	TTC34	-1,192
TMEM167B	-0,83	GPRC5B	-1,405	PLCL2	-1,15
MAP2K4	-0,78	CDK6	-1,334	BCL2	-1,117
CIR1	-0,76	SRP14-AS1	-1,291	KLHL5	-1,113
NPEPL1	-0,71	CYP51A1	-1,275	CDK6	-1,097
LUCAT1	-0,70	CYP1A2	-1,264	ATP5E	-1,083
ARMC5	-0,58	PIWIL4	-1,25	CISH	-1,059
ZNF529	0,50	MTRF1	-1,244	SNORA2A	-1,028
FOXJ3	0,51	SHROOM1	-1,24	SPTA1	-1,011
KIDINS220	0,52	IPO7	-1,232	XPO7	-0,975
CGGBP1	0,54	ZNF34	-1,231	DACT1	-0,958
TMBIM6	0,57	ZBTB16	-1,221	CASC4	-0,915
UBQLN2	0,57	ADRBK2	-1,218	ZDHHC20	-0,887
SF3B2	0,59	RHBDD1	-1,218	UPP1	-0,871
ST6GAL1	0,60	DYNC1I2	-1,199	CLEC4D	-0,871
NOLC1	0,61	IL20RB	-1,198	SNORA34	-0,866
TMEM248	0,63	SLC36A4	-1,184	MKRN1	-0,863
TPP1	0,63	EPHX1	-1,178	TNS1	-0,858
ANKRD40	0,64	MGAT5	-1,177	WASF1	-0,851
SETD1A	0,66	PDK4	-1,154	EEF1E1	-0,847
SF3B3	0,67	PGAP1	-1,153	SNORD66	-0,831
NCOR2	0,68	SREBF2	-1,144	SOCS2	-0,825
TES	0,69	CLPB	-1,135	TRIM58	-0,823
NOL11	0,70	KLF11	-1,133	ZNF101	-0,816
EIF2B5	0,70	CERS6	-1,125	SPA17	-0,801
RBM10	0,71	RSPO3	-1,121	TSPAN5	-0,788
YLPM1	0,71	LTF	-1,116	DCAF6	-0,77
ELMO2	0,72	SGMS2	-1,106	FCHSD2	-0,763
XPO5	0,72	FADS1	-1,098	RAB2B	-0,763
RFC1	0,73	PTX3	-1,095	PAGE2	-0,762
RANGAP1	0,73	PEA15	-1,094	MARCH8	-0,75
BOP1	0,74	NID1	-1,094	SNORD25	-0,731
ZCCHC3	0,74	KLHL8	-1,094	PAGE2B	-0,729
DCAF7	0,75	NRIP1	-1,086	NLRC4	-0,722
SPECC1L	0,75	PTPN9	-1,084	MCPH1	-0,718
ILF3	0,75	NECAP1	-1,084	FIS1	-0,706
SSRP1	0,75	CCDC149	-1,077	MKRN7P	-0,704
WBP11	0,76	DIAPH2	-1,066	SESN3	-0,69
LRBA	0,76	DHCR24	-1,065	DCAF12	-0,677
HNRNPA3	0,77	EIF3B	-1,062	OR2W3	-0,676
DDX23	0,77	AGAP3	-1,057	CD58	-0,667
HNRNPM	0,77	SRD5A1	-1,055	ITGAX	-0,654
INTS9	0,78	EMILIN2	-1,054	NKAPD1	-0,651
CASP2	0,79	HAUS7	-1,052	SPAG16	-0,628
SLC9A9	0,79	ZNF93	-1,05	CPNE3	-0,619

NRIP1	0,79	STS	-1,045	YPEL2	-0,612
SQLE	0,80	ZNF594	-1,04	RSL1D1	-0,572
STRIP1	0,80	BTBD3	-1,035	ZNF654	-0,571
GPATCH8	0,80	GPR125	-1,034	ARL6IP6	-0,444
PUM1	0,80	TBC1D2B	-1,006	LAT2	0,497
CLSTN1	0,81	DACT1	-1,006	ELL	0,537
ZMAT3	0,81	UPP1	-1,005	PTPN23	0,538
USP11	0,82	EGR1	-1,002	AARS2	0,56
YWHAZ	0,82	ETFDH	-1,001	CHMP2A	0,571
TRIM26	0,83	THRAP3	-1	AVIL	0,582
PLOD3	0,86	SNORD52	-0,996	PIK3CD	0,602
EHMT2	0,86	TLR7	-0,976	EIF2B5	0,611
RRP1B	0,86	CAMTA2	-0,974	GALM	0,615
LRCH3	0,87	SNTB1	-0,974	DDX39A	0,646
RNGTT	0,91	RUNX1	-0,969	KMT2A	0,658
TRIM28	0,91	SORT1	-0,96	HNRNPC	0,666
SNRNP70	0,93	PLXNB2	-0,954	MXD3	0,671
EWSR1	0,93	ZNF623	-0,954	PRR12	0,683
WBSCR16	0,93	ZHX3	-0,95	MID1IP1	0,683
SLC25A22	0,93	GPATCH8	-0,948	NXF1	0,685
ZNF777	0,95	ZMYM3	-0,942	SAFB2	0,686
PPP5C	0,98	ZNRF2	-0,94	TRIM26	0,687
NUP210	0,99	CIAPIN1	-0,937	DAZAP2P1	0,692
CNOT4	1,02	DPYSL2	-0,928	KLF12	0,695
ABCF1	1,09	CASP3	-0,922	ND4	0,7
ANKRD36BP1	1,09	CYBB	-0,921	TTC3	0,713
DOCK7	1,13	RAB3GAP1	-0,92	TAF5	0,719
DDX24	1,23	HK3	-0,917	HDAC10	0,722
HSPB1	1,37	HECTD4	-0,911	SLC3A2	0,727
		GLUD2	-0,907	POLRMTP1	0,733
		INTS9	-0,903	ZNF646	0,735
		DNM1L	-0,902	CCDC142	0,739
		MIOS	-0,895	TRIM28	0,739
		LOXL3	-0,895	HNRNPH3	0,75
		BIRC6	-0,89	ZNF767P	0,75
		SOWAHC	-0,89	SNRPN	0,75
		FLNB	-0,885	FGD3	0,751
		CSTF3	-0,883	TPRN	0,751
		MGA	-0,882	LY9	0,759
		BOP1	-0,88	OGFOD2	0,763
		SOCS2	-0,877	KRTCAP2	0,77
		PKM	-0,875	PARVG	0,784
		TMEM237	-0,87	ZNF512	0,792
		EGR2	-0,866	HM13	0,81
		ATP1A4	-0,864	PILRA	0,811
		FCN1	-0,863	ARHGDIA	0,812
		GIMAP8	-0,859	DDX24	0,812
		CD300E	-0,857	NOP2	0,838
		NLRC4	-0,856	IGF2R	0,847
		RPS24	-0,853	KLHL22	0,852
		GNPAT	-0,852	KIF21B	0,859
		LAIR1	-0,849	SRRT	0,86

MTSS1	-0,849
SLC46A2	-0,848
EDARADD	-0,847
UBAP2L	-0,843
CCDC6	-0,841
PTGIR	-0,839
CCR2	-0,839
NCKAP1L	-0,836
DAGLB	-0,833
TNFSF13	-0,826
CLEC4D	-0,825
TRAF3IP1	-0,82
ENO1	-0,818
PUM1	-0,817
ZFHX3	-0,817
SCARB2	-0,817
STAC3	-0,816
CD163	-0,815
SLC27A3	-0,814
RNMT	-0,812
ATP2A2	-0,811
GNL3L	-0,807
TBC1D8	-0,804
MRPL15	-0,801
ALAS1	-0,8
SLC30A7	-0,799
CSF1R	-0,799
UBE3A	-0,789
TAPT1	-0,789
AGPAT5	-0,789
TRIAP1	-0,786
FOXI2	-0,783
FGD6	-0,781
SMG8	-0,781
VDAC1	-0,781
PDLIM5	-0,78
PBRM1	-0,779
MS4A7	-0,775
CNIH4	-0,774
RIN2	-0,771
SNX30	-0,771
ACO2	-0,769
KPNA6	-0,769
PTPRA	-0,768
FCHSD2	-0,762
EHMT2	-0,761
NUDT21	-0,761
LARP1	-0,757
IMPAD1	-0,755
GNS	-0,752
IL15	-0,749
CCSER2	-0,748
PLEKHO1	0,861
NOV	0,879
ALOX5AP	0,892
KCTD7	0,903
TNFAIP8L2	0,907
CYHR1	0,929
RUNX1-IT1	0,929
BANF1	0,939
ADRM1	0,958
KISS1	0,961
PPP1R11	0,963
LETM2	0,963
AMD1P3	0,968
BCOR	0,983
DLEU2L	0,993
MIA	0,996
TJAP1	0,999
TAF9P3	1,004
GRID2IP	1,011
TCRA	1,013
ABCG1	1,021
WDR20	1,025
SGTA	1,027
PBXIP1	1,028
RBM14	1,038
CTU1	1,05
FBRSL1	1,061
CDC14A	1,094
CLP1	1,115
ASPSCR1	1,118
IRF3	1,131
EWSR1	1,132
GNL1	1,14
PRMT7	1,15
DMAP1	1,197
KIAA2026	1,273
ZNF114P1	1,403
INO80C	1,575
MYH7B	1,638

SLC26A6	-0,747
RAD50	-0,743
PIGF	-0,742
FUT4	-0,742
BCKDHA	-0,741
TMEM30A	-0,741
LNPEP	-0,736
LTA4H	-0,735
IL27RA	-0,735
RIC1	-0,733
TRIM44	-0,732
TNS3	-0,732
QSER1	-0,732
CDYL2	-0,731
SLC30A1	-0,729
ALDH3B1	-0,726
SON	-0,726
USP19	-0,725
EFTUD2	-0,724
MPDU1	-0,724
IARS2	-0,722
NAGA	-0,72
LRBA	-0,719
KIAA1598	-0,718
CECR1	-0,717
SNX29	-0,716
AP4E1	-0,714
CCDC22	-0,713
TAOK3	-0,71
ANKS1A	-0,705
NECAP2	-0,7
COPA	-0,699
TPP1	-0,698
ATP13A3	-0,694
MMGT1	-0,692
RPS27L	-0,683
RCC2	-0,679
TDG	-0,677
HNRNPA3	-0,677
DDX23	-0,674
NOL11	-0,674
SPECC1L	-0,674
CENPQ	-0,674
HPS5	-0,671
MAFB	-0,671
EIF2B5	-0,668
DPYD	-0,667
LRP1	-0,664
MBIP	-0,662
VTA1	-0,66
RRAS	-0,66
KLHL42	-0,659

SETD1A	-0,656
DCAF13P3	-0,651
ZC3H7A	-0,649
CELF2	-0,649
SNRPE	-0,648
CTNNB1	-0,648
QTRTD1	-0,639
DBI	-0,638
GBAS	-0,638
MRPS35	-0,636
ZNF614	-0,633
KIAA2018	-0,631
RBBP4	-0,63
NOLC1	-0,63
ATAD2B	-0,627
ZBTB21	-0,62
ITGAM	-0,619
DDX10	-0,613
KCTD12	-0,61
YWHAG	-0,609
ZNF106	-0,608
NDFIP1	-0,605
FLNA	-0,605
NUP160	-0,605
UGP2	-0,603
EXT2	-0,603
STARD7	-0,601
TAB2	-0,6
DHX30	-0,6
NUP62	-0,599
AGPS	-0,598
ATP13A1	-0,593
KLF9	-0,592
TARDBP	-0,591
COX6C	-0,587
SPG11	-0,582
VPS36	-0,579
ZNF260	-0,569
DDX54	-0,567
APLP2	-0,564
CLSTN1	-0,562
YWHAB	-0,562
GCOM1	-0,56
EDRF1	-0,559
BZW2	-0,558
NAB1	-0,558
PSAP	-0,556
IL17RA	-0,556
DYNC1H1	-0,555
RECK	-0,553
CITED2	-0,552
NCOR2	-0,55

RNF138	-0,55
RNF219	-0,549
WASL	-0,547
ZZZ3	-0,546
GGCT	-0,546
IREB2	-0,544
PANK3	-0,544
SLC26A2	-0,541
ARFGEF2	-0,539
GRN	-0,539
DCAF7	-0,539
KIDINS220	-0,535
AGGF1	-0,535
PRKAB2	-0,533
FBXO21	-0,532
AEBP2	-0,531
KPNA3	-0,53
PDP1	-0,529
MTHFD2	-0,528
ITGAV	-0,527
CPOX	-0,527
FBXO30	-0,524
FBXW11	-0,522
EEA1	-0,522
MBD2	-0,521
BRD1	-0,519
ZNF721	-0,516
HECTD1	-0,508
DDX21	-0,508
ATP8A1	-0,503
SNX14	-0,501
TMEM263	-0,5
BRCC3	-0,498
TNFAIP8	-0,497
TRAPPC13	-0,497
SACS	-0,495
FOXP1	-0,491
C2CD5	-0,488
PLEKHB2	-0,487
HTATSF1	-0,486
USP34	-0,482
RAB12	-0,481
NUS1	-0,475
PHC3	-0,475
G3BP1	-0,47
EIF4G2	-0,47
ZC3H15	-0,468
ZFAND5	-0,465
SKIV2L2	-0,465
CCAR1	-0,461
MIER3	-0,456
VPS13C	-0,45

NCOA6	-0,449
EFR3A	-0,444
SOCS4	-0,435
HNRNPA1	-0,432
UFL1	-0,404
RNF6	-0,404
MTDH	-0,394
REEP5	-0,384
HIST1H3A	0,518
AQP5	0,528
NDEL1	0,531
HIST1H2BN	0,541
YIPF3	0,543
SLC5A2	0,547
RAF1	0,553
LEPREL2	0,556
RAB11FIP1	0,561
HIST1H3E	0,576
MXD3	0,587
SLC25A51	0,591
RABL2B	0,593
HIST3H3	0,597
ELL	0,613
FCGR2C	0,616
TSEN34	0,621
CDRT15L2	0,625
CPPED1	0,633
RPL13AP17	0,634
ABTB1	0,645
HIST1H3J	0,646
CREB5	0,646
NOTCH1	0,649
TMEM71	0,653
SOD2	0,654
STK40	0,657
H3.Y	0,657
TMEM88	0,661
GLT1D1	0,665
CTBS	0,666
TNFSF14	0,681
LUCAT1	0,682
FRAT1	0,683
IDS	0,683
POU5F1	0,685
COQ10A	0,686
H3F3A	0,687
ACTR3C	0,691
NADK	0,693
GTF2IRD2B	0,699
EBLN2	0,708
KRTAP5-11	0,713
KAZN	0,721

PHF20L1	0,724
KIAA1257	0,726
CBX7	0,744
AVIL	0,747
NCF4	0,771
OSER1	0,777
CCL16	0,778
GCSAM	0,783
CYBC1	0,785
TCRA	0,786
SYNPO	0,791
PPP1R14B	0,793
NPIPA5	0,794
LHX4-AS1	0,815
ALPK3	0,818
SPATA2L	0,825
SYNE4	0,825
RARA	0,84
BCL2L12	0,84
SLC15A4	0,842
DAZAP2P1	0,842
DGAT2	0,847
OTOP1	0,853
GNAI3	0,855
BSDC1	0,856
MAPKAPK5	0,856
HNRNPC	0,864
TRMT5	0,87
MTRNR2L2	0,875
MAK	0,888
KLF12	0,889
MTX1	0,897
HSPB9	0,898
HN1	0,898
GABARAPL1	0,901
MRVI1	0,902
KLHL22	0,905
TAGLN2	0,915
LITAF	0,916
JAK1	0,919
NPL	0,928
ELF2	0,935
LIMK2	0,939
ABCA1	0,945
SLC2A1	0,947
NAMPT	0,947
MAFF	0,97
MESP1	0,983
SLC45A4	0,991
ALKBH8	0,992
GNL1	1,004
DLEU2L	1,006

PPP1R15A	1,038
TNFRSF10C	1,043
BCL3	1,064
SPG7	1,067
PSENEN	1,086
RGP1	1,087
PHF12	1,092
NMB	1,108
TSPYL2	1,116
TREM1	1,119
ARHGAP15	1,159
DENND5B	1,163
PEBP4	1,167
HNRNPCL1	1,23
TBP	1,231
NDUFA6-AS1	1,231
NPIPB5	1,237
AZGP1	1,242
LOXL1	1,253
SPACA3	1,306
ARHGEF39	1,341
ZNF746	1,463
ABCG1	1,513

Supplementary Table 3: List of upstream regulators obtained with IPA for each analysis

MDD vs. NON-MDD at baseline

Upstream Regulator	Molecule Type	Activation z-score	p-value of overlap	Target Molecules in Dataset
TP53	transcription regu	1,702	1,75E-04	BOP1,CASP2,HSPB1,MAP2K4,NCOR2,NOLC1,PPP5C,RANGAP1,RFC1,RRP1B,SQLE,STAMBP,TPP1,TRIM28,UBQLN2,XPO5,ZMAT3

Non-MDD after vs. before surgery

Upstream Regulator	Molecule Type	Activation z-score	p-value of overlap	Target Molecules in Dataset
GATA1	transcription regu	-0,478	5,46E-05	ALOX5AP,BCL2,CDK6,ITGAX,NOP2,PBXIP1,SLC3A2,SPTA1,TRA
IL31	other		1,71E-04	CDK6,CISH,SOCS2
KIT	transmembrane r		3,04E-04	BCL2,CISH,ITGAX,SOCS2,SPTA1
IL31RA	transmembrane r		3,28E-04	CISH,SOCS2
DNMT3L	transcription regu		4,91E-04	IGF2R,SNRPN
HIPK2	kinase	1,067	6,18E-04	ALOX5AP,AVIL,BCL2,XPO7
HISTONE	group		7,55E-04	BCL2,CDK6,KISS1

MDD after vs.before surgery

Upstream Regulator	Molecule Type	Activation z-score	p-value of overlap	Target Molecules in Dataset
dihydrotestosterone	chemical - endoge	-0,584	4,25E-07	ABCA1,ABCG1,ACO2,ALAS1,ATP2A2,AZGP1,BCL2,CASP3,CCL16,CTNNB1,CYBB,DBI,DDX54,DHCR24,EGR1,ENO1,GRN,H3C12,MAFF,MAK,NOTCH1,PEA15,RAD50,RPS24,RUNX1,SLC2A1,SORT1,SRD5A1,SREBF2,TAPT1,ZBTB16

TP53	transcription regı	0,507	1,60E-06	ACO2,BCL2,BCL3,BOP1,CASP3,CERS6,CITED2,CPOX,CSF1R,CTNNB1,CYP51A1,DBI,DHCR24,DNM1L,EGR1,EGR2,EIF4G2,EPHX1,GRK3,IL17RA,IL27RA,IPO7,IREB2,ITGAV,LIMK2,MAFB,MAP4,MRPL15,MTDH,NAB1,NAMPT,NCOR2,NLRC4,NOLC1,NOTCH1,PDLIM5,PDP1,PEA15,PIGF,PKM,PLEKHB2,PLXNB2,POU5F1,PPP1R15A,PRKAB2,PSAP,PTPRA,RAD50,RAF1,RBBP4,RPS27L,RUNX1,SLC2A1,SOCS2,SOD2,SON,TAGLN2,TBP,TDG,TNFAIP8,TNFRSF10C,TPP1,TRIAP1,TRIM44,UPP1,VDAC1,YWHAG
dexamethasone	chemical drug	-3,103	2,84E-06	ABCA1,ALAS1,AQP5,ATP13A3,ATP2A2,BCKDHA,BCL2,BCL3,BZW2,CASP3,CCR2,CD163,CELF2,CLEC4D,COPA,CSF1R,CTNNB1,CYBB,CYP1A2,CYP51A1,DGAT2,DHCR24,DNM1L,DPYD,EDARADD,EGR1,EGR2,EMILIN2,ENO1,FLNB,GGCT,GNPAT,GNS,GRK3,GRN,IDS,IL15,ITGAM,ITGAV,KLF9,LAIR1,LITAF,LRP1,MAFF,MBD2,MBIP,NAGA,NCOR2,NLRC4,NRIP1,PDK4,POU5F1,PSAP,PTX3,RAD50,RARA,RCC2,SCARB2,SLC26A2,SLC26A6,SLC2A1,SLC30A7,SLC5A2,SOCS2,SOD2,SORT1,TNFSF13,TNFSF14,UBE3A,UGP2,ZBTB16

CSF2	cytokine	-1,472	4,19E-06	[illegible]163,CSF1R,CYBB,EGR1,EGR2,GRK3,IL15,ITGAM,PPP1R15A,RARA,RBBP4,SLC2A1,SLC30A7,SNTB1,SOCS2,SOD2,SREBF2,TNFSF14,TREM1,UPP1
CSF1	cytokine	-2,340	6,26E-06	BCL2,CD163,CSF1R,CTNNB1,CYBB,CYP51A1,DHCR24,EGR1,ENO1,ITGAM,ITGAV,MAFB,PKM,RUNX1,SLC2A1,SREBF2
CD24	other	-2,478	8,44E-06	ATP13A3,DIAPH2,KLHL22,NRIP1,PPP1R15A,RAD50,RAF1,RASA1,SLC30A1,SPG11,VPS13C
GFER	enzyme		9,30E-06	CYP1A2,DNM1L,FUT4,POU5F1
desmopressin	biologic drug	-1,698	1,34E-05	ARFGEF2,CTNNB1,ENO1,FLNA,H3-3A/H3-3B,LRBA,PUM1,SORT1,VDAC1,YWHAB
IL5	cytokine	-2,924	1,55E-05	BCL2,BCL3,CCR2,CITED2,CYP1A2,DDX21,EGR1,EGR2,ENO1,ITGAM,ITGAV,LIMK2,MTDH,PKM,SLC2A1,SNTB1,SOCS2,UPP1
RPTOR	other	-1,689	1,62E-05	CDK6,CSF1R,CTNNB1,CYP51A1,DHCR24,ENO1,ITGAM,PKM,SLC2A1,SREBF2
IRF8	transcription regu	-2,246	1,95E-05	CASP3,CCR2,CSF1R,CYBB,EGR1,EGR2,IL15,IL17RA,ITGAM,JAK1,MAFB,ZFHX3
FLT1	kinase	-1,000	2,04E-05	APLP2,BSDC1,DHCR24,FLNA,G3BP1,NOTCH1,RBBP4,SLC27A3,VDAC1

lipopolysaccharide	chemical drug	-1,050	2,41E-05	ABCA1,ABCG1,AEBP2,AQP5,ATP2A2,AZGP1,BCL2,BCL3,CASP3,CCR2,CD163,CITED2,CLEC4D,CSF1R,CTNNB1,CYBB,CYP1A2,DHCR24,EGR1,EGR2,ENO1,GGCT,GNPAT,H3-3A/H3-3B,IL15,IL17RA,IL20RB,IREB2,ITGAM,ITGAV,LIMK2,LITAF,LRBA,LRP1,LTA4H,LTF,MAFB,MAFF,MTDH,MTSS1,NAMPT,NDFIP1,NID1,NOTCH1,PBRM1,PDK4,PKM,PPP1R15A,PTX3,RARA,SLC2A1,SLC30A1,SLC5A2,SOCS2,SOD2,STS,SYNPO,TLR7,TNFSF13,TNFSF14,TREM1,YWHAG,ZFHX3
ABCA1	transporter	0,128	2,79E-05	ABCA1,ABCG1,CYP51A1,DGAT2,NAMPT,SLC2A1,SREBF2
ERK	group	-1,757	2,82E-05	ABCA1,BCL2,CTNNB1,DPYSL2,EGR1,EGR2,ITGAM,ITGAV,MAFB,MAFF,PDK4,POU5F1,RECK,SLC2A1,SORT1,TNFAIP8
OSCAR	other	0,816	4,44E-05	BCL2,CCR2,ITGAM,RRAS,TLR7,TNFSF14
LDL	complex	0,571	5,24E-05	ABCA1,ABCG1,ATP2A2,BCL2,CASP3,CCR2,CTNNB1,CYBB,EGR1,IL17RA,ITGAV,LRP1,NAMPT,RARA,SOD2,SREBF2
ciprofloxacin	chemical drug	0,632	6,27E-05	ACO2,ALDH3B1,EMILIN2,GRN,IMPAD1,PLXNB2,PSAP,RAB11FIP1,TLR7,VDAC1
MYC	transcription regu	-2,749	6,36E-05	[illegible]K6,CLPB,CSF1R,CTBS,CTNNB1,DBI,DDX21,DDX54,EFTUD2,EGR1,EGR2,ENO1,EXT2,FLNA,GNPAT,H3-3A/H3-3B,HNRNPA1,IPO7,IREB2,ITGAM,MAP4,M
FN1	enzyme	-1,622	6,78E-05	APLP2,BCL2,BSDC1,CDK6,DHCR24,FLNA,G3BP1,ITGAM,ITGAV,POU5F1,RBBP4,RECK,SLC2A1,VDAC1

BCR (complex)	complex	0,585	6,86E-05	BCL2,CASP3,CDK6,CTNNB1,EGR1,JAK1,KLF9,LAIR1,NAMPT,SLC2A1,ZBTB16
bisphenol A	chemical - endoge	-0,640	7,72E-05	ALAS1,AQP5,BCL2,CASP3,CITED2,DHX30,DNM1L,EGR1,ENO1,PKM,POU5F1,SRD5A1
TGFB1	growth factor	-0,416	7,92E-05	ABCA1,ABCG1,ATP13A3,BCL2,BCL3,CASP3,CCR2,CD163,CELF2,CITED2,CSF1R,CTNNB1,CYBB,DDX21,EGR1,EGR2,EHMT2,ENO1,EXT2,FLNA,FLNB,GNL1,GNPAT,GNS,GPRC5B,HNRNPC,IL15,ITGAM,ITGAV,KLF9,KPNA3,KPNA6,LIMK2,LITAF,LOXL1,LRBA,LTA4H,MAP4,MGAT5,MTHFD2,MXD3,NAB1,NAMPT,NOTCH1,NUP62,PDLIM5,PIGF,PKM,PTX3,RARA,RASA1,RECK,RUNX1,SLC2A1,SLC5A2,SOD2,TAB2,TARDBP,UBE3A
FOXO4	transcription regu	2,608	9,07E-05	CYP51A1,DHCR24,FLNB,ITGAM,NAMPT,SLC2A1,SOD2,SREBF2,ZFAND5
APP	other	0,074	9,27E-05	ABCA1,ABCG1,ACO2,APLP2,ATP1A4,BCL2,CASP3,CDK6,CITED2,CSF1R,CTNNB1,CYBB,DBI,DNM1L,DPYSL2,EGR1,ENO1,FLNA,HNRNPA1,IL15,ITGAM,LRP1,MAFB,MAFF,NAMPT,PEA15,PKM,PSENEN,SNX14,SOD2,SREBF2,TLR7,TPP1,VDAC1,YWHAB,ZFHX3
PRKAG3	other		1,04E-04	ABCA1,ALAS1,CELF2,EGR1,IL15,JPT1,MAP4,NAMPT,NRIP1,PPP1R14B,SLC2A1,UGP2

miR-1-3p (and other miF	mature microRNA	1,063	1,05E-04	BCL2,CPOX,EHMT2,H3-3A/H3-3B,KLHL42,LRP1,MTHFD2,MTX1,PLEKHB2,RABL2B,RNF138,SHTN1,TAGLN2
D-tubocurarine	chemical drug		1,21E-04	EGR1,LITAF,PTPRA,UBE3A
EPAS1	transcription regu	0,191	1,29E-04	BCL2,CCR2,CITED2,CYP51A1,DGAT2,ENO1,ITGAV,MAFF,NAMPT,NOTCH1,POU5F1,SLC2A1,SOD2,UGP2
IL4	cytokine	-2,777	1,48E-04	ABCA1,ALAS1,BCL2,BCL3,BOP1,CASP3,CD163,CDK6,CELF2,CITED2,CPOX,CSF1R,CYBB,DYNC1H1,EEA1,EGR2,EPHX1,FADS1,FLNA,FLNB,IL15,IL27RA,ITGAV,JAK1,KCTD12,KIDINS220,LRP1,LTA4H,MAFB,MAP4,MTDH,RAD50,SLC2A1,SOCS2,SON,VPS13C,ZZZ3
NFkB (complex)	complex	0,015	1,59E-04	ABCG1,AQP5,ATP2A2,BCL2,BCL3,CCR2,CTNNB1,CYBB,EGR1,GNPAT,IL15,ITGAM,ITGAV,LITAF,MTSS1,NAMPT,NOTCH1,NUP62,PEA15,PTX3,RRAS,RUNX1,SLC2A1,SOD2,TNFAIP8,TNFRSF10C,TNFSF14
LIPE	enzyme	0,692	1,59E-04	ABCA1,CITED2,EGR1,ELL,H3-3A/H3-3B,NRIP1,PDK4,RARA,SCARB2,YWHAG
Immunoglobulin	complex	0,835	1,61E-04	ACO2,BCL2,BCL3,CCR2,CDK6,CSF1R,CTNNB1,DPYSL2,EGR1,EGR2,ENO1,FLNB,HK3,KCTD12,MAFF,MTHFD2,NAMPT,PDK4,SLC2A1,SOD2,TLR7,TNFSF13,UGP2,ZBTB16

filgrastim	biologic drug	-2,074	1,72E-04	ATP13A3,CD163,CLEC4D,CSF1R,CYBB,DPYD,EDARADD,EGR2,EMILIN2,ENO1,GNS,ITGAM,KLF9,LAIR1,MAFF,MBIP,NCOR2,NLRC4,RAD50,SLC26A6,SORT1
TSPO	transmembrane r		2,41E-04	BCL2,SOD2,VDAC1
desmosterol	chemical - endoge		2,41E-04	ABCA1,ABCG1,DHCR24
TNFSF11	cytokine	-0,395	2,43E-04	[illegible]GRN,IL15,ITGAM,ITGAV,MAFB,RRAS,SLC2A1,SOD2,ZNF260
triamcinolone acetonide	chemical drug	-0,087	2,43E-04	BCL2,CCDC6,EGR1,FUT4,KLF9,RUNX1,SOCS2,SOD2,TNFAIP8,TRA,TSPYL2,ZBTB16,ZFAND5
NPC1	transporter		2,71E-04	ABCA1,ABCG1,CCR2,CDYL2,CYP51A1,EGR1,SRD5A1,SREBF2
NRG1	growth factor	-0,839	2,85E-04	ABCA1,BCL2,CASP3,EGR1,EGR2,FLNA,G3BP1,ITGAM,ITGAV,LIMK2,NCOR2,NOTCH1,POU5F1,RUNX1,SLC2A1
Ige	complex	-1,374	3,00E-04	ALAS1,ATP2A2,BCL2,BCL3,EGR1,EGR2,EIF4G2,IL15,ITGAV,NCF4,PEA15,PTPN9,PTX3,RUNX1,TLR7,TMEM71,TNFSF14
IL6	cytokine	-1,126	3,39E-04	ABCA1,ALAS1,ATP2A2,BCL2,BCL3,CASP3,CCR2,CD163,CSF1R,CTNNB1,CYBB,CYP1A2,EGR1,EGR2,IL15,ITGAM,ITGAV,JAK1,LTF,MRVI1,NAMPT,NOTCH1,POU5F1,PPP1R15A,RASA1,SLC5A2,SOCS2,SOD2,STS,TLR7
TCL1A	transcription regu		3,43E-04	CLEC4D,CYBB,IL17RA,ITGAM,LTF,MAFB,SCARB2,TLR7
ZNF326	transcription regu		3,78E-04	RAD50,SLC36A4

CACYBP	other		3,78E-04	BCL2,CTNNB1
HIF1A-AS1	other		3,78E-04	BCL2,CASP3
cyclo(iso-Asp-GR)-LLIIKL/	chemical reagent		3,78E-04	BCL2,CASP3
camptothecin	chemical drug	0,756	4,14E-04	BCL2,BIRC6,CASP3,CDK6,DENND5B,DNM1L,EGR2,FCHSD2,H3-3A/H3-3B,HPS5,IL17RA,LITAF,MAFF,PTX3,RARA,RASA1,SLC30A1,SMG8,SOD2,SOWAHC,SPG7,TAGLN2,TNS3,TPP1,TSPYL2
TNF	cytokine	-1,094	4,34E-04	ABCA1,AQP5,ATP2A2,BCKDHA,BCL2,BCL3,BTBD3,CASP3,CCR2,CD163,CERS6,CITED2,CSF1R,CTNNB1,CYBB,DBI,EGR1,EGR2,FADS1,FUT4,GNAI3,GNL1,GPRC5B,GRN,HK3,IL15,ITGAM,ITGAV,JAK1,LITAF,LUCAT1,MAFF,MTDH,NAMPT,NCOR2,NID1,NOTCH1,NRIP1,PBRM1,PEBP4,PKM,PLXNB2,PPP1R15A,PTX3,RARA,SLC2A1,SLC5A2,SOCS2,SOD2,SYNPO,TBC1D8,TLR7,TNFAIP8,TNFSF14,TNS3,TREM1,YWHAG
APOA1	transporter	1,387	4,50E-04	ABCA1,CCR2,CYBB,ITGAM,SLC2A1,SOD2
brimonidine	chemical drug		5,63E-04	BCL2,CTNNB1,GNAI3
BDNF	growth factor	1,032	5,99E-04	BCL2,CASP3,EGR1,EGR2,FLNA,FLNB,NMB,PDLIM5,PLXNB2,PTPN9,REEP5,SOD2,SYNPO,TBC1D8,TPP1,YWHAG

IFNG	cytokine	-0,181	6,16E-04	ABCA1,ADA2,ANKS1A,ATP2A2,AZGP1,BCL2,BCL3,CASP3,CCR2,CD163,CERS6,CSF1R,CTNNB1,CYBB,CYP1A2,EGR1,EGR2,ENO1,FCN1,GNL1,GPRC5B,IL15,IL17RA,IREB2,ITGAM,ITGAV,KCTD12,LUCAT1,MAFF,MTSS1,NAMPT,NOTCH1,PEA15,PKM,PTX3,RAB12,SLC2A1,SLC5A2,SOCS2,SOD2,SORT1,SREBF2,TLR7,TNFSF13,TREM1,ZBTB16
NCSTN	peptidase	1,457	6,24E-04	CSF1R,GRN,NCF4,NOTCH1,PSENEN
lithium chloride	chemical drug	-0,479	6,46E-04	BCL2,BCL3,CTNNB1,CYP51A1,IDS,NOTCH1,POU5F1,PTX3,RARA
OGA	enzyme	-0,229	6,48E-04	ACO2,AZGP1,BCL2,CDK6,CIAPIN1,COX6C,CREB5,CSF1R,CTNNB1,DAGLB,EGR2,FLNA,FLNB,HK3,JAK1,MTX1,SOCS2,SREBF2,TBP
mifepristone	chemical drug	1,137	6,70E-04	[illegible],ITGAM,KLF11,LRP1,LTF,NPL,PTX3,RARA,SLC2A1
resveratrol	chemical drug	1,434	6,93E-04	BCL2,CASP3,CD163,CDK6,CSF1R,CTNNB1,CYBB,CYP1A2,EGR1,LITAF,NOTCH1,NRIP1,PDK4,PPP1R15A,SOD2
PSEN1	peptidase	2,579	7,54E-04	ABCG1,ACO2,ATP1A4,ATP2A2,CASP3,CTNNB1,CYP51A1,DBI,DNM1L,DPYSL2,EGR1,ENO1,LRP1,NOTCH1,PEA15,PKM,PSENEN,VDAC1
FOXO3	transcription regu	1,812	7,79E-04	BCL2,DNM1L,EGR1,EGR2,FLNB,GABARAPL1,ITGAM,JAK1,MTHFD2,MXD3,NAMPT,NOLC1,PPP1R15A,RASA1,SOD2,SREBF2
Pkg	group	0,404	7,83E-04	BCL2,CTNNB1,EGR1,SOD2

APR-246	chemical drug		8,05E-04	BCL2,LIMK2,MAP4,PEA15,YWHAG
tanespimycin	chemical drug	0,529	8,15E-04	BCL2,CDK6,HNRNPA1,HNRNPC,RAF1,SLC2A1,SNRPE,SOD2,SREBF2
SP1	transcription regu	-1,325	8,60E-04	[illegible] CYP51A1,DHCR24,DIAPH2,EGR1,FLNA,IL15,ITGAM,ITGAV,PKM,POU5F1,RARA,RECK,R
propofol	chemical drug	0,132	9,09E-04	BCL2,CASP3,CYBB,EGR1,SOD2
ESRRA	ligand-dependent	-2,742	9,51E-04	ALAS1,ATP2A2,CYP51A1,ENO1,ETFDH,LTF,NRIP1,PDK4,PDP1,PKM,RARA
sodium arsenite	chemical drug	-0,555	9,88E-04	BCL2,CDK6,EGR1,ENO1,LITAF,RAF1

Supplementary Table 4 : List of target genes following ChEA database for TP53, NR3C1 and RELA transcription factors.

TP53 target genes from ChEA	NR3C1 targe genes from ChEA	RELA target genes from ChEA
AARS2	AACS	ABCA1
ABCB6	ABCD3	ABCB1
ABCB9	ABCG2	ABCB4
ABCF2	ABHD11	ABCB9
ABHD1	ABHD15	ABCC6
ABHD5	ABLIM3	ABHD2
ABL1	ACAA2	ABI1
ACAD11	ACACA	ABR
ACADVL	ACAP2	ABTB2
ACAP1	ACBD6	ACHE
ACBD6	ACHE	ACSS1
ACSF2	ACSL5	ADAM19
ACTA2	ACSM3	ADCK3
ACTB	ADAM10	ADCK4
ACTBL2	ADAMTSL2	ADORA1
ACTN4	ADAT2	ADRBK2
ADAM15	ADI1	AGER
ADAM19	ADRA1A	AGTRAP
ADIPOR1	AGFG1	AHCTF1
ADPGK	AGPAT3	AHNAK
ADRB2	AGPAT5	AKAP13
AEBP2	AGTPBP1	AKR1B1
AEN	AKAP12	AKR1C1
AGAP1	AKAP8	ALCAM
AGL	AKR1B1	ALDH3A1
AGPS	ALDH18A1	ALOX12
AIMP1	ALDH1A1	ALOX12B
AKAP10	ALDH7A1	ALOX5
AKAP13	ALOXE3	ALPK1
AKAP9	ANKRD28	AMACR
ALAD	ANKRD50	AMH
ALDH3A1	APBB2	ANGPT1
ALOX5	APCDD1	ANKLE2
AMBRA1	APOL3	ANKRD28
AMZ2	APOL6	ANKRD9
ANKHD1	AQP1	ANXA7
ANKHD1-EIF4EBP3	ARFGEF2	AP1S3
ANKRD11	ARG2	AP2A1
ANKRD12	ARID1B	AP2B1
ANKRD17	ARIH2	AP4M1
ANKZF1	ARL3	APBB2
AP4S1	ARL4A	APOBEC3A
APAF1	ARL4C	APOBEC3B
APBB2	ARL9	APOE

APBB3	ARMC7	APP
APITD1	ARRDC5	AR
APOLD1	ASAP2	ARF1
ARCN1	ASXL2	ARFRP1
ARHGAP22	ATAD2B	ARHGAP27
ARHGAP26	ATF1	ARHGEF2
ARHGEF3	ATIC	ARHGEF40
ARID3A	ATOH8	ARID1A
ARID3B	ATXN1	ARID2
ARL2	ATXN10	ARL4A
ARL8B	AZIN1	ARL5B
ARSG	AZU1	ARL6IP5
ASB16	B3GALT2	ARNT2
ASCC3	B4GALT1	ASAP1
ASTN2	B4GALT5	ASB6
ATAD2B	B9D1	ASPH
ATF3	BANP	ASS1
ATG4A	BBS2	ATG13
ATG9A	BCAS4	ATG16L2
ATL1	BCAT1	ATOX1
ATN1	BCL6	ATP9A
ATP2A2	BLK	ATR
ATRIP	BOD1	ATXN2L
ATXN7L3	BTBD11	AZIN1
AXL	BTBD2	B2M
B3GAT2	BTG1	B3GAT3
BACH1	BTN3A1	B4GALT5
BAI2	BTNL3	BACE1
BAX	C1D	BACH1
BBC3	CACNA1C	BAIAP2L2
BBS9	CAMK1D	BAX
BCAS3	CAND1	BBC3
BCL2L1	CBR4	BCAT1
BEST4	CBS	BCL2
BIN2	CBX4	BCL2A1
BLOC1S2	CBX8	BCL2L1
BRE	CCDC122	BCL2L11
BTG2	CCDC93	BCL3
CAMK2D	CD163	BCL9L
CAMK4	CD320	BFSP1
CAPN12	CD48	BHLHE40
CAPNS1	CD82	BIN3
CAPZA2	CD83	BIRC2
CARS	CD9	BIRC3
CC2D1A	CDC14C	BLNK
CCDC47	CDC42EP3	BLOC1S1
CCDC51	CDHR3	BMI1
CCDC57	CDK1	BNIP1

CCDC85B	CDK20	BNIP3
CCND2	CDK5RAP2	BOD1
CCNG1	CDKN3	BRCA2
CCNG2	CEBPB	BRE
CD180	CELA1	BRI3BP
CD70	CELF5	BTG3
CD79A	CENPF	BTK
CDC42BPB	CETP	C1QTNF6
CDC42EP3	CFDP1	C1S
CDC73	CHD2	C2
CDKL1	CHD9	C3
CDKN1A	CHEK2	C4BPA
CDS2	CHML	CA13
CEND1	CHST4	CALD1
CEP68	CIDEC	CANT1
CES2	CIITA	CAPNS1
CGREF1	CITED2	CARD14
CHAD	CLASP1	CASP10
CHD9	CLDN10	CASP4
CHI3L2	CLDN19	CAV1
CHMP4A	CLEC16A	CBR3
CHMP7	CLEC2L	CBX5
CHPF2	CLIC5	CCDC107
CHRM4	CLP1	CCDC115
CLEC18B	CMTM8	CCDC124
CLK3	CNOT6L	CCDC25
COL11A2	COL23A1	CCDC57
COL13A1	COPS8	CCDC64
CORT	CPEB2	CCDC94
COX5A	CPEB4	CCDC97
COX6A1	CPNE5	CCL17
COX6B2	CPSF6	CCL19
CPA5	CR1L	CCL2
CPEB2	CROCC	CCL23
CR1L	CRY1	CCL28
CREB3	CSGALNACT2	CCL5
CRELD1	CSNK1G3	CCND2
CRLF1	CSRP2BP	CCND3
CROCC	CTAGE1	CCNG1
CSPG4	CTDSPL	CCNL1
CSRP1	CTNNB1	CCR5
CTF1	CTSD	CCR7
CTSW	CUX1	CD209
CUEDC1	CXCL12	CD274
CWF19L1	CXCL2	CD38
DCAF12	CXCR5	CD3G
DCK	CXCR6	CD40
DCP1A	CYB5D2	CD40LG

DCP1B	CYTH1	CD44
DCST2	DACT1	CD48
DCUN1D3	DAP	CD55
DDB2	DAP3	CD59
DDX41	DCT	CD63
DDX42	DDHD1	CD69
DEAF1	DDX3X	CD70
DENND2D	DEGS2	CD74
DENND5B	DENND3	CD80
DERL3	DENND4A	CD83
DGKZ	DFNB31	CD86
DHDH	DGAT2	CDC14B
DHRS1	DIP2B	CDC37
DHX37	DIRC3	CDC42EP4
DLG4	DIS3L	CDC42EP5
DNAJA1	DLEU7	CDH23
DNAJC22	DLG5	CDK12
DOK3	DNAJC10	CDK17
DPEP3	DNAJC24	CDK6
DRAM1	DNAJC6	CDKN1A
DRAP1	DOCK5	CDX1
DSE	DOK7	CEACAM1
DSN1	DPYSL2	CEBPD
DVL2	DSCR9	CFL2
E2F7	DTNA	CFLAR
EBNA1BP2	DUSP1	CHD2
EDC3	DUSP4	CHDH
EDEM1	DUSP5	CHI3L1
EDF1	DYNLT1	CHST15
EEA1	DYSF	CKS1B
EFNA4	EBF1	CLCF1
EFR3A	ECE1	CLDN16
EGFL7	EDN1	CLDN4
EI24	EDNRA	CLIC1
EIF3K	EEPD1	CLIC4
EIF3L	EFCAB2	CLTA
EIF5A	EFR3A	COTL1
ELK3	EID1	COX4I1
ENO2	EID3	CPD
ENOSF1	EIF3H	CPLX2
ENTPD6	EIF4E3	CR2
EPHB2	ELMO2	CREB1
EPHB4	ELP3	CREB3
EPS8L2	ENPP4	CS
ERBB2	ENTPD6	CSAD
ERBB2IP	EPB41L2	CSF1
EVC	EPHA4	CSRP2
EVI5L	EPRS	CTSB

EXOC3	EPS8	CTSS
FARP2	ERCC1	CXCL1
FAS	ERGIC1	CXCL10
FASN	ETAA1	CXCL2
FBRS	EXPH5	CXCL5
FBXL14	EZR	CXCL9
FBXL18	FAT3	CXCR1
FBXL19	FBXL18	CXCR2
FBXO22	FBXO11	CXCR5
FBXW4	FBXW12	CXXC5
FCHO2	FCRL2	CYB561
FDXR	FEM1B	CYB561D2
FER	FGF4	CYB5A
FGFR1	FGGY	CYBA
FHL2	FIP1L1	CYFIP1
FIBCD1	FKBP8	CYTH1
FIBP	FNBP1L	CYTH4
FITM2	FOXO1	DCLRE1A
FLAD1	FOXP1	DCTN4
FOXN3	FRY	DCTPP1
FRMD4A	FUCA1	DDA1
FRMD8	GADD45B	DDR1
FSCN1	GALC	DDX26B
FTL	GAST	DDX47
FTSJ3	GFRA2	DEFB4A
FUT10	GGCX	DENND3
GABARAP	GJD4	DENND4A
GADD45A	GLI2	DHPS
GAL3ST4	GNB4	DHRS7B
GAN	GNG7	DHX9
GAS6	GOLPH3	DIAPH2
GATC	GOPC	DICER1
GBA2	GOSR2	DIRC2
GBE1	GPAM	DLG4
GCC2	GPM6B	DNAJB6
GDF15	GPN2	DNASE1L2
GHDC	GPR124	DOCK10
GLB1L	GPR35	DPP3
GLG1	GRAMD3	DPP4
GLIPR2	GRB10	DPYD
GLRX2	GRID2	DSTN
GLTSCR1	GSN	DTX2
GML	GSS	DUSP1
GNG7	GSTM3	DUSP10
GNRH2	GTDC1	DUSP22
GPATCH1	GUCY2GP	DUSP3
GPC1	GYPC	DUSP5
GPC2	H2AFY	DUSP6

GPC3	3F3C	DYRK2
GPHN	AAO	E2F3
GPR180	ACE1	EBI3
GPS2	AO1	ECT2
GPX1	AUS3	EDN1
GRB7	DAC8	EFEMP2
GRIN2C	EATR3	EFHC2
GSN	ECTD1	EGFR
GYS1	ECTD2	EGR1
H3F3A	IP1	EHBP1
HAAO	IPK2	EHD2
HAUS6	IST1H1D	EIF2AK3
HCRT	IST1H2AC	EIF4A2
HDAC11	IST1H2BG	ENG
HELZ	IST1H4H	ENO1
HGFAC	LCS	ENO2
HNRNPUL1	MGN2	ENO3
HOXA3	MHB1	EPHA1
HOXA4	PCAL1	EPS8
HOXA5	SBP1	ERBB2
HPDL	SCB	ERF
HS3ST3B1	FT52	ESPL1
HSD17B8	GFBP3	ETS2
IGF2BP2	KZF2	F11R
IKBIP	NTS10	F2RL3
IKZF3	NTU	F8
IL17RC	PO5	FARS2
INPP5B	RF8	FAS
INPP5K	RS2	FASLG
IPO4	OSD2	FASTKD5
IQCE	PH3	FBXO46
IRF2BP2	CNG1	FBXW11
ISG20	CNJ2	FCER2
ISYNA1	CNMB3	FCGBP
ITGAM	CNQ1OT1	FCGRT
ITPA	CTD12	FCHO1
JUN	IAA0125	FCHSD2
KBTBD6	IAA1467	FECH
KCNH4	IAA1841	FFAR2
KCNN4	IT	FKBP15
KCTD18	LF12	FLNA
KDM6A	LF6	FLOT2
KIAA0100	LHL29	FOS
KIAA1324	LHL3	FOSL2
KIF2A	LHL38	FOXK1
KISS1R	LRD1	FOXP4
KLHL12	YNU	FRMD4A
KLHL25	ARGE	FSCN1

LACTB	LDLR	FSTL3
LAPTM5	LDOC1L	FTH1
LASP1	LHFP	G3BP1
LATS2	LHPP	G6PD
LBX1	LIG1	GABPB1
LGR6	LIMK1	GADD45B
LIMA1	LMCD1	GALT
LMNA	LMO7	GATA3
LMNB1	LMX1B	GBP1
LOXHD1	LPAR1	GCH1
LRFN4	LPHN2	GCLC
LRP1	LRRC1	GCLM
LRRC25	LRRC27	GCNT1
LRRC47	LUC7L2	GFRA1
LRRC8B	MAFB	GGT1
LSG1	MAN2B1	GLI1
LSM3	MAOA	GLRX
LTBR	MAP2K3	GNAI2
LYST	MARCO	GNAL
M6PR	MBIP	GNB2L1
MAD1L1	MBOAT2	GNRH2
MAK16	MCM6	GPBP1
MAMDC4	MIOS	GPR108
MAN1C1	MKLN1	GPR137
MANEAL	MKNK1	GPR56
MAP4	MLF1	GPX4
MAP4K4	MLLT3	GRAMD1A
MAST4	MMAB	GRB7
MCM4	MPHOSPH9	GRIN2A
MCPH1	MRGPRG	GRK5
MDK	MRM1	GRN
MDM2	MRPL1	GSDMD
MDP1	MRPL46	GSN
MED26	MRPS11	GSTP1
MED4	MRPS33	GTF2E1
METAP1	MSI2	GZMB
MFSD2A	MSN	HAMP
MGAT2	MTSS1	HAS1
MGRN1	MYOM1	HAVCR1
MICALL1	MYSM1	HBZ
MINPP1	N4BP1	HCST
MKLN1	NANOS3	HDGF
MLLT10	NBPF15	HELLS
MOAP1	NCAM1	HGF
MRPS23	NCKIPSD	HIF1A
MRPS26	NCOA6	HIVEP2
MRPS31	NDRG1	HLA-A
MSI2	NDUFA10	HLA-B

MTF1	NDUFB1	HLA-G
MTHFD1L	NEBL	HMGCS1
MTMR10	NEDD4L	HMGN1
MUTYH	NEK1	HMGXB4
MYO1C	NELL2	HMOX1
MYO1E	NFIC	HNRNPF
N4BP2	NHLRC3	HOXA3
NAALADL1	NIPAL2	HPSE
NAB2	NPHP3	HSD17B8
NACC2	NPTXR	HSP90AA1
NAP1L4	NR3C1	HSPA1A
NAV3	NR3C2	HSPA1L
NEAT1	NR5A2	HSPG2
NEGR1	NRCAM	ICAM1
NEK7	NRG1	ICOS
NEURL1B	NRP1	IDO1
NEURL4	NSMAF	IER2
NF1	NSUN7	IER3
NFKBIZ	NT5C2	IER5
NHLH2	NTRK2	IFI44L
NHS	NUB1	IFIH1
NKX3-1	OGDH	IFNAR2
NOTCH1	OGFOD2	IFNG
NOTCH3	OLFM1	IFT20
NPM2	OPLAH	IGFBP2
NR4A2	OSBPL5	IGSF3
NRBP2	OSTC	IKBKB
NRG1	P2RY1	IKBKE
NSFL1C	PAICS	IKZF3
NSUN2	PANX1	IL15
NUDCD2	PCBD1	IL15RA
OAT	PCDH9	IL17A
OC90	PCNXL2	IL17F
OR52K2	PDGFC	IL18R1
OSBPL3	PDHB	IL1B
OSM	PDIA6	IL1RN
P2RY2	PDS5A	IL23A
PAIP1	PDZD2	IL27
PAK6	PELI1	IL2RA
PALLD	PER2	IL32
PANX1	PEX7	IL4I1
PARP12	PHF3	IL6
PC	PHLDA1	IL7
PCBD2	PIAS1	INHBA
PCNA	PID1	INO80C
PCNXL2	PITRM1	IQCD
PDGFC	PKDCC	IRF1
PDLIM7	PKIA	IRF2

PDSS2	PKNOX1	IRF2BP2
PGD	PLA2G2C	IRF4
PGPEP1	PLB1	IRF7
PHC1	PLEKHA2	ITFG3
PHF12	PLEKHJ1	ITGB1
PHF14	PLIN3	ITGB2
PHF20	PMVK	ITPRIP
PHF23	POLR1D	ITSN2
PHLDA3	PON2	JMY
PHLDB1	PPARGC1B	JUN
PHPT1	PPIAL4G	JUNB
PI4KA	PPM1A	KCNJ15
PKD2L1	PPP1CC	KCNK5
PKN2	PPP1R12B	KCNN2
PLCB2	PPP1R2	KDM2A
PLCG2	PPP3CA	KDM6B
PLEKHF2	PPP3CC	KIAA0430
PLEKHG1	PRIMA1	KISS1
PLEKHG7	PRKAG3	KLF10
PLK2	PRKCE	KLF3
PLK3	PRKDC	KLF4
PLXDC1	PRKX	KLF5
PLXNB2	PROC	KLHL21
PMAIP1	PRPS1	KLHL5
PNPLA2	PRSS38	KLK3
POLE3	PSAT1	KLRAP1
POLR2I	PTAR1	KRT15
POU3F1	PTK2B	KRT18
PPDPF	PTPN4	KRT3
PPM1D	PTPRJ	KTN1
PPP4R1	PTPRM	LAMB2
PRC1	PTPRO	LAMB3
PRDM1	PTPRS	LBR
PREB	PUM2	LCN2
PRKAG2	PWWP2A	LDLR
PRKAR1B	QPCT	LEF1
PRKCE	RAB11FIP2	LENG9
PRKDC	RAB1A	LFNG
PRODH	RAB3IP	LGALS1
PRR14	RAB7A	LGALS3
PRR5	RAD23B	LHFPL2
PRR5-ARHGAP8	RALB	LMNA
PRRT3	RANBP2	LPGAT1
PSMC5	RAPGEF1	LPXN
PSMD3	RAPGEF2	LRG1
PTCH2	RAPH1	LSR
PTEN	RASGRP1	LTA
PTP4A1	RAVER2	LTB

PTPN6	RBBP6	LTBP4
PTPRA	RBM26	LTF
PTPRM	RCC2	LYRM4
PUF60	RCOR3	LZIC
PUM1	REPS2	MADCAM1
PVRL1	RERG	MAFF
PVT1	RFC3	MAFG
QRFP	RFX8	MAML2
RAB10	RGS18	MANBAL
RAB1A	RHOB	MAP2K3
RABGGTA	RHOU	MAP3K11
RANGAP1	RIC8B	MAP3K8
RAP2B	RILPL1	MAP4K1
RASL11A	RLF	MAP7
RBBP4	RNF144A	MAPK14
RBPJ	RNF220	MARCKS
RCC2	RNGTT	MASTL
RCCD1	RPL21P44	MBP
RCE1	RPL29	MCM7
RCN1	RPL38	MDK
RDH14	RPL39L	MED13
REC8	RPS29	MED15
RECK	RPS6KC1	METTL2B
REV3L	RQCD1	MGLL
RGP1	RSRC1	MID1IP1
RGS12	RTKN2	MID2
RHOA	RUNX3	MITD1
RHOBTB2	RUSC2	MKLN1
RHPN2	RYBP	MLLT11
RILP	SAMD4A	MLLT6
RILPL1	SARDH	MMD
RING1	SARS	MMP1
RPL21	SBNO1	MMP9
RPL23	SCARNA20	MRPS24
RPL36AL	SCOC	MT3
RPLP2	SDHAF2	MTHFR
RPP25	SEC24C	MTMR2
RPS19	SEC31A	MUC1
RPS27L	SEL1L3	MUC2
RPTOR	SENP8	MX1
RRAGA	SERBP1	MYADM
RRAGD	SERINC2	MYB
RRBP1	SERP2	MYC
RRM2	SETBP1	MYH9
RRM2B	SFRP2	MYLK
RRP1B	SGK1	MYO1G
RSAD1	SH3BP4	MZF1
RSBN1L	SHOX2	NAB2

RSF1	SLA	NAMPT
RTKN	SLC25A24	NANS
RTN3	SLC2A1	NAV1
RUNX3	SLC2A10	NBEAL1
RXRB	SLC2A6	NBN
S100A2	SLC37A1	NCAM1
SAC3D1	SLC39A10	NCOA4
SACS	SLC45A4	NCOA7
SAE1	SLC46A3	NDE1
SAMD12	SLC9A4	NDST1
SARS	SLC9A8	NDUFA7
SBF2	SLTM	NDUFB7
SCAMP1	SMARCA2	NDUFV2
SCAND1	SMTN	NEK6
SCARF1	SNX13	NEK8
SCARNA8	SNX8	NEU1
SCRIB	SOBP	NFAT5
SDF2	SOCS1	NFATC2IP
SDPR	SOCS3	NFIC
SEC61A1	SORCS3	NFKB1
SELM	SORL1	NFKB2
SERTAD1	SP110	NFKBIA
SERTAD3	SP9	NFKBIB
SESN1	SPAG16	NFKBID
SESN2	SPATA2	NFKBIE
SEZ6	SPRY2	NFKBIZ
SF3A3	SPRY4	NGF
SGCB	SQRDL	NHLRC2
SIAH2	SRC	NINJ1
SIRPB2	SRGAP1	NIPA1
SKI	SRGAP2	NLRP2
SLC12A2	SRPK1	NMNAT1
SLC12A4	ST3GAL1	NOD2
SLC16A3	ST3GAL5	NOTCH2NL
SLC25A15	ST8SIA4	NPRL2
SLC25A22	STK24	NR3C1
SLC25A45	STK25	NR4A2
SLC2A8	STK3	NRCAM
SLC30A1	STK32C	NRG1
SLC34A3	STK39	NSFL1C
SLC35A4	STMN1	NT5M
SLC35E4	STX4	NTRK3
SLC38A10	STXBP5	NUB1
SLC38A2	SULT1B1	NUMB
SLC39A7	SUPT3H	NXT1
SLC43A2	SYK	NXT2
SLC4A11	SYNRG	OLA1
SMAD3	SYT2	OPN1SW

SMAGP	SYTL2	OPRD1
SMARCB1	TAB2	ORM1
SMARCD2	TAOK3	OXTR
SMARCD3	TBL1XR1	P2RY6
SMARCE1	TCF7L2	PABPC1
SMEK1	TES	PAFAH2
SMOC1	TFF2	PAN2
SMTN	TFF3	PANX1
SMU1	TGDS	PAPPA
SMYD3	TGFBI	PARD3
SNAP29	TGFBR2	PARK7
SNHG12	TGIF1	PARP1
SNORA16A	TGOLN2	PARP4
SNORA21	THADA	PATL1
SNORA27	TIAM2	PAWR
SNORA44	TIGD2	PAX8
SNORA52	TIPARP	PBX2
SNORA61	TLE1	PCDH1
SNORD12	TMEM138	PDCD2L
SNORD12B	TMEM170B	PDCD5
SNORD12C	TMEM189	PDE10A
SNORD99	TMEM2	PDE4D
SNX15	TMTC1	PDE6D
SPAG9	TMTC3	PDE7A
SPATS2	TNFRSF13B	PDGFB
SPEN	TNFSF13B	PDLIM7
SPR	TNS3	PDZD2
SQLE	TOX	PER1
SRA1	TOX2	PFDN4
SRC	TP53BP2	PFKP
SRD5A1	TPD52	PFN1
SRXN1	TPRA1	PGK1
SSBP4	TRERF1	PGLYRP1
STAG3	TRIB1	PHLDA1
STARD3	TRMT11	PHLDB1
STARD4	TRPC1	PHLDB2
STAT5B	TRPS1	PHLPP2
STAT6	TSEN2	PI3
STAU2	TSPAN5	PIGF
STEAP3	TSPYL2	PIK3AP1
STK17A	TTC12	PIK3C2B
STK24	TTC32	PIK3CA
STK25	TTC39B	PIM1
STOM	TTC7B	PJA2
STRN3	TULP4	PLA2G4A
SUPT6H	TWISTNB	PLA2G4C
SUSD1	TXNRD3	PLAU
SUV420H2	UBASH3A	PLCD1

SYNC	UBASH3B	PLEC
SYNE1	UBE2E1	PLEKHG2
TBC1D22B	UBE2E2	PLEKHG3
TBC1D4	UBE2NL	PLEKHG6
TBCB	UBE2T	PLK3
TBCK	UBL3	PLXNB2
TCN2	UBR2	PNKD
TCP11L1	UCK2	POLR2A
TCTE1	UFC1	POMC
TCTEX1D4	ULK4	PPAPDC2
TESK2	URB2	PPIF
TFDP1	URM1	PPP1R14B
TGDS	USP13	PPP1R15A
TGFBI	USP30	PPP1R15B
TGFBR3	USP39	PPP5C
TGM1	VAMP8	PPP6R3
TKT	VAPA	PPTC7
TLE1	VAV2	PQBP1
TLN1	VPS35	PRCP
TM9SF1	VPS37D	PRDM1
TMEM106C	VPS8	PRF1
TMEM194A	WAC	PRKAA1
TMEM217	WDHD1	PRKAB2
TMEM30A	WIPF1	PRKACA
TMEM44	WLS	PRKCD
TMEM80	WNK2	PRKCH
TMUB2	WNT7B	PRMT1
TNFRSF10A	WSB1	PRPF39
TNFRSF10B	WWC1	PRRG4
TNFRSF10C	XPR1	PRRT2
TNFRSF10D	YPEL1	PSEN1
TNRC18	YPEL5	PSMA2
TOE1	YWHAQ	PSMB9
TP53INP1	ZAK	PSME1
TPM2	ZC3H7B	PSME2
TPX2	ZCCHC11	PTAFR
TRANK1	ZDHHC17	PTEN
TRIAP1	ZDHHC18	PTGDS
TRIM22	ZEB2	PTGER4
TRIM32	ZFP36L2	PTGIR
TRIM5	ZFYVE26	PTGS2
TRIT1	ZHX2	PTMA
TRNAU1AP	ZMAT3	PTP4A2
TRPC1	ZMYND8	PTPN1
TRPC2	ZNF133	PTPN12
TRRAP	ZNF333	PTPN13
TSGA10	ZNF341	PTPN2
TSPAN14	ZNF438	PTS

TSSK4
TXNDC15
TYMS
UBA52
UBE2G2
UBE2J1
UBE2Q2
UBN2
UBR5
UBTF
UBXN2A
UGCG
ULK2
UNC45A
UNC5B
UPF1
USP10
USP4
UST
UTF1
VAC14
VAMP4
VAPA
VAV2
VENTX
VOPP1
VPS13C
WDR43
WDR54
XPC
XPO7
YRDC
YWHAH
ZAK
ZBED4
ZBTB7B
ZC3H7B
ZFAND2B
ZMAT3
ZNF205
ZNF250
ZNF337
ZNF397
ZNF506
ZNF572
ZNF594
ZNF654
ZNF79
ZNF462
ZNF615
ZNF639
ZNF667
ZNF674
ZNF703
ZNF791
ZWINT
PTTG1IP
PTX3
PWWP2A
PYCARD
QKI
RAB11FIP5
RAB21
RAB27A
RAB30
RAB3IP
RALY
RAPGEF3
RAPH1
RARG
RASA2
RB1CC1
RBBP4
RBKS
RBM14
RBM17
RBM23
RBM47
RCAN1
RCHY1
RDH16
RDH5
REL
RELA
RELB
REPIN1
REV3L
RFFL
RFTN1
RFX2
RFX5
RHCG
RHOF
RHOG
RILPL2
RIPK2
RNASE4
RNF126
RNF19A
RNF31
RPH3AL
RPL23A
RPS2
RPS27A

ZNFX1
ZSWIM4

RPS28
RTEL1
RUFY1
RUSC1
S100A10
S100A13
S100A2
S100A4
S100A6
SAR1B
SCRN2
SDC4
SDCBP
SEC24A
SECISBP2
SELP
SEMA4C
SENP2
SENP3
SERPINA1
SERPINA3
SERPINB1
SERPINB8
SERPINH1
SF3B1
SH2D3A
SH3BGRL
SH3BP4
SHC1
SIAH1
SIX5
SKIL
SKP2
SLC11A2
SLC16A1
SLC16A5
SLC25A12
SLC25A37
SLC25A45
SLC29A1
SLC2A5
SLC2A6
SLC30A7
SLC31A1
SLC36A4
SLC39A10
SLC3A2
SLC48A1

SLC5A2
SLC6A6
SLC7A5
SLC9A8
SLFN12
SLITRK5
SLPI
SMCHD1
SMG1
SMPD1
SMPD2
SMURF1
SNAI1
SNAP25
SNAPC1
SNUPN
SOD1
SOD2
SOX13
SP3
SP7
SPAG9
SPATA2L
SPECC1
SPI1
SPP1
SPPL2A
SPSB1
SQSTM1
SRC
SRCAP
SRGAP1
SRGN
SRRT
SS18
SSSCA1
ST3GAL1
ST6GAL1
ST6GALNAC1
ST7
ST8SIA1
STARD3NL
STAT1
STAT5A
STAT5B
STAT6
STK39
STK40

STX10
STX11
STX1A
STX4
STX8
SUB1
SUPT16H
SUPV3L1
SWAP70
SYNGR2
SYNPO
SYS1
SYT2
TAF1B
TAF1D
TAF4B
TANK
TAP1
TAPBP
TBC1D10A
TBC1D12
TBL1X
TBL1XR1
TBRG4
TCF19
TCF7L2
TCN2
TEF
TERT
TESK2
TFCP2
TFE3
TFEC
TFF1
TFF3
TFG
TGM1
TGM2
THAP6
THBS1
THOC1
TICAM1
TIMM17B
TJP3
TLCD1
TLE1
TLR2
TLR9

TMBIM1
TMBIM4
TMEM102
TMEM105
TMEM173
TMEM50A
TMTC2
TNF
TNFAIP1
TNFAIP2
TNFAIP3
TNFRSF10B
TNFRSF1B
TNFRSF4
TNFRSF9
TNFSF10
TNFSF13
TNFSF13B
TNIP1
TOM1
TP53
TP53I3
TPCN1
TPD52
TPM1
TPM4
TPMT
TPPP3
TPRA1
TPT1
TRAF1
TRAF2
TRAF4
TRAPPC10
TRAPPC3
TREM1
TRIM16L
TRIM25
TRIM44
TRIM47
TRIM69
TRIM8
TRIOBP
TRIP4
TRPC1
TSG101
TSNARE1
TSPAN14

TWISTNB
TXNL1
TXNRD1
UBB
UBE2M
UBE2Z
UBN2
UBOX5
UBQLN4
UCP2
UGGT1
UGP2
UMPS
UNC13D
UNKL
UPF3B
UPP1
UQCRC2
USP49
USP54
UTRN
UXS1
VEGFC
VEZF1
VHL
VIM
VPS53
VRK1
WDR11
WDR7
WDR89
WNT10B
WRAP53
WWC1
WWP1
XIAP
YY1
ZBED3
ZBTB1
ZBTB25
ZC3H12A
ZCCHC8
ZDHHC24
ZFHX2
ZFHX3
ZFP36L1
ZFP91
ZFX

ZHX2
ZMIZ2
ZNF101
ZNF33A
ZNF33B
ZNF436
ZNF438
ZNF467
ZNF574
ZNF706
ZNF823
ZNRF1

Supplementary Table 5: Multivariate regression with adjustment for Age, Sex and BMI.

TP53: adjustment for Age, Sex and BMI

Synthèse de la Régression; Variable Dép. : MADRSTOT (Obespsy2019stats.sta)
R= ,80746311 R²= ,65199668 R² Ajusté = ,60228191
F(4,28)=13,115 p<,00000 Err-Type de l'Estim.: 4,5905

N=33	Bêta	Err-Type de Bêta	B	Err-Type de B	t(28)	niveau p
OrdOrig.			15,03103	9,464956	1,588071	0,123500
AGE	-0,060805	0,112653	-0,03834	0,071035	-0,539755	0,593633
SEX	0,008784	0,113270	0,21902	2,824231	0,077549	0,938738
IMC	-0,039525	0,113212	-0,05613	0,160760	-0,349126	0,729607
ChEA TP53M0	0,807600	0,114238	59,51306	8,418368	7,069429	0,000000

NR3C1: adjustment for Age, Sex and BMI

Synthèse de la Régression; Variable Dép. : MADRSTOT (Obespsy2019stats.sta)
R= ,79994758 R²= ,63991613 R² Ajusté = ,58847557
F(4,28)=12,440 p<,00001 Err-Type de l'Estim.: 4,6695

N=33	Bêta	Err-Type de Bêta	B	Err-Type de B	t(28)	niveau p
OrdOrig.			19,56816	9,554568	2,048042	0,050038
AGE	-0,113469	0,115858	-0,07155	0,073056	-0,979385	0,335777
SEX	-0,082737	0,114227	-2,06294	2,848100	-0,724320	0,474878
IMC	-0,039322	0,115187	-0,05584	0,163564	-0,341376	0,735369
ChEA NR3C1 M0	0,803898	0,116813	59,59298	8,659359	6,881916	0,000000

RELA: adjustment for Age, Sex and BMI

Synthèse de la Régression; Variable Dép. : MADRSTOT (Obespsy2019stats)
R= ,79869932 R²= ,63792061 R² Ajusté = ,58619498
F(4,28)=12,333 p<,00001 Err-Type de l'Estim.: 4,6825

N=33	Bêta	Err-Type de Bêta	B	Err-Type de B	t(28)	niveau p
OrdOrig.			21,28979	9,56420	2,22599	0,034240
AGE	-0,122716	0,116457	-0,07738	0,07343	-1,05375	0,301006
SEX	-0,029401	0,114949	-0,73308	2,86610	-0,25578	0,799993
IMC	-0,081075	0,114806	-0,11512	0,16302	-0,70619	0,485907
ChEA RELA M0	0,801226	0,116939	71,28411	10,40390	6,85167	0,000000
